# Comparative Evaluation of Deep Learning Object Detectors for Real-Time Parking Occupancy Detection Under Variable Lighting Conditions

**DOI:** 10.3390/s26175329

**Published:** 2026-08-22

**Authors:** Fernando G. Yunganina Mamani, Guver L. Ccori Coarite, Jhon A. Chambi Vilca, Angel Rosendo Condori-Coaquira, David Mamani-Pari, Milton Edward Humpiri-Flores, Esteban Tocto-Cano

**Affiliations:** Professional School of Systems Engineering, Faculty of Engineering and Architecture, Universidad Peruana Unión, Juliaca 21101, Peru; gary.yunganina@upeu.edu.pe (F.G.Y.M.); guverccori@upeu.edu.pe (G.L.C.C.); alexander.chambi@upeu.edu.pe (J.A.C.V.); angelrc2@upeu.edu.pe (A.R.C.-C.); davidmp@upeu.edu.pe (D.M.-P.); milton.humpiri@upeu.edu.pe (M.E.H.-F.)

**Keywords:** object detection, smart parking, YOLOv8, transfer learning, computer vision, real-time, vehicle monitoring, deep learning

## Abstract

Efficient parking space management in urban settings represents a growing challenge owing to the sustained increase in the vehicle fleet. This study presents a comparative evaluation of five object detection architectures —YOLOv8s, YOLOv11s, YOLOv12s, RT-DETR-L and Faster R-CNN—applied to real-time intelligent vehicle occupancy monitoring under variable lighting conditions. The models were trained via transfer learning on a custom dataset of 1463 source images (21,944 annotated instances; expanded to 3511 files and 52,664 instances through offline augmentation of the training subset; three classes: *free*, *occupied* and *unavailable*) captured on a university campus located in Juliaca (Puno region), Peru, at 3824 m a.s.l. under daytime and nighttime clear-sky conditions from a single fixed-camera viewpoint. Each architecture was evaluated in ten independent experiments. Six dataset partitioning schemes of increasing strictness—a random control (R0) plus five leakage-controlled partitions—were evaluated. Under the strictest scheme (D3), simultaneously disjoint in acquisition date and camera viewpoint and therefore the most rigorous generalization estimate obtained in this study, accuracy ranges from mAP@0.5:0.95 of 0.9325 for Faster R-CNN to 0.8763 for YOLOv11s. Under the random partitioning conventionally applied to fixed-camera datasets, the same five architectures fell within 0.0055 of one another, all above 0.985, and their ranking was essentially inverted (Spearman ρ=−0.80). The differences in computational efficiency across architectures were statistically significant (H=47.06, p<0.001). YOLOv8s was the fastest of the four non-dominated architectures under the disjoint partition and was selected in 73.3% of weightings, although it ranked fourth in accuracy; its recommendation therefore rests on computational efficiency under a real-time constraint, whereas deployments that prioritize accuracy are better served by Faster R-CNN. The integrated system YOLOv8s + ByteTrack + FastAPI + Next.js 14 achieved per-slot accuracies of 87.5% and 91.8% under daytime and nighttime clear-sky conditions, respectively, using 1395 observations collected in a single university parking lot. For YOLOv8s, the transition from random to disjoint partitioning costs 0.1085 in mAP@0.5:0.95 (0.9913 to 0.8828), indicating that the near-saturated performance obtained under random partitioning substantially reflects the memorization of a fixed spatial configuration rather than generalization. The results support the feasibility of single-stage CNN architectures for intelligent parking monitoring in high-altitude Andean university environments under the evaluated acquisition conditions.

## 1. Introduction

Efficient parking space management represents an increasingly significant operational challenge in urban environments, where the sustained growth of vehicle fleets places constant pressure on infrastructures with limited capacity [1,2]. In response to this scenario, intelligent parking systems have been extensively studied for their ability to optimize vehicle allocation using real-time monitoring [3]. The imbalance between parking demand and space availability gives rise to a phenomenon known as *cruising*, which is defined as vehicles circulating in search of available parking space [4]. This behavior increases search times and contributes to traffic congestion, particularly during peak-demand periods [2]. It has also been reported that the distance traveled during this search can reach up to 2.7 times the optimal route, resulting in higher fuel consumption and increased CO_2_ emissions [4]. In quantitative terms, reducing the average per-vehicle cruising time by three minutes at a facility with 15 monitored slots and 50 vehicle arrivals per day—conservative estimates consistent with the university environment studied in this work—would eliminate approximately 37 km of unnecessary vehicle travel daily (15 km/h campus speed) and avoid approximately 4.5 kg of CO_2_ emissions per day, corresponding to roughly 1.6 tonnes per year at a single facility (assuming an emission factor of 120 g CO_2_/km for a mixed passenger-vehicle fleet [4]). While this projection illustrates the potential scale of the impact of small monitored installations, the benefit scales proportionally with the number of facilities and users equipped with real-time occupancy data. In this context, dynamic pricing models based on real-time occupancy data have been shown to improve parking infrastructure utilization [5].

Traditional systems based on physical sensors installed in each parking slot have scalability and maintenance limitations that hinder their adoption in large-scale parking facilities [3]. Ultrasonic sensors are sensitive to adverse weather conditions, inductive sensors provide a limited detection range, and conventional optical systems often generate false positives owing to lighting variations, shadows, and reflections. Their implementation also requires modifications to the existing infrastructure, increasing installation, maintenance, and hardware replacement costs.

As an alternative, computer vision-based systems enable the monitoring of multiple parking slots using conventional cameras without requiring modifications to the physical infrastructure [6]. This approach has gained momentum owing to the maturity of convolutional object detectors capable of real-time inference on commodity hardware, and has been applied to parking monitoring across edge and cloud configurations [7].

Among the architectures designed for this type of application, the YOLO family stands out because it provides a balance between accuracy and speed of inference. YOLOv8 incorporates a decoupled detection head and an *anchor-free* design [8]; YOLOv11 introduces C3k2 blocks—a cross-stage partial structure with two bottleneck convolutions that refines gradient flow—together with partial self-attention (PSA) modules in the neck [9]; and YOLOv12 introduces *area-attention*, a spatial self-attention mechanism restricted to local rectangular partitions of the feature map rather than computed globally, substantially reducing the quadratic cost of full self-attention while maintaining spatial context, with optional FlashAttention support for memory-efficient computation [10]. Both YOLOv11 and YOLOv12 descriptions were drawn from Ultralytics technical documentation and an arXiv preprint, respectively, whichv are standard sources for rapidly evolving open-source frameworks but are not subject to the same peer-review process as traditional journal publications. In contrast, the RT-DETR proposes an end-to-end *transformer*-based architecture that eliminates the need for non-maximum suppression (NMS), enabling real-time detection [11]. Faster R-CNN employs a two-stage framework based on *Region Proposal Networks* (RPNs) and ROIAlign, achieving high localization accuracy at the expense of greater computational requirements [12]. Despite these advances, comparative evaluations that systematically analyze one-stage CNN detectors, *transformer*-based models, and two-stage detectors specifically applied to parking monitoring under variable environmental conditions remain limited [13,14].

This study presents a comparative evaluation of five object detection models—YOLOv8s, YOLOv11s, YOLOv12s, RT-DETR-L, and Faster R-CNN for parking occupancy monitoring under variable lighting conditions and quantifies how strongly the conclusions of such a comparison depend on the dataset partitioning protocol. The main contributions of this study are as follows.

1. **Quantification of the distortion introduced by random partitioning in fixed-camera parking datasets.** A perceptual-hashing audit established that 50.2% of the test images under a conventional random split share a 15-min acquisition window with the training images. Six partitioning schemes of increasing strictness—a random control (R0) plus five leakage-controlled partitions—were then defined and evaluated, isolating the temporal and geometric components of the leakage and their interaction. The effect is not confined to inflated figures: moving from random to fully disjoint partitioning costs 0.1085 in mAP@0.5:0.95 for YOLOv8s, widens the spread between the best and worst architecture from 0.0013 to 0.0562, expands the Pareto front from one architecture to four, and essentially inverts the ranking of the five models (Spearman ρ=−0.80). Therefore, a leakage-affected protocol does more than overstate performance; it removes the information that would allow architectures to be distinguished at all, and can reverse the resulting recommendation.
2. **A model selection framework was evaluated using both protocols.** Pareto dominance, a composite scoring function, and a Monte Carlo sensitivity analysis over 10,000 weight vectors were applied to the same five architectures under random and disjoint partitioning. The comparison shows that the framework is uninformative under the former—any weighting returns the same model and becomes discriminative only under the latter, where the selection frequency of the leading model decreases from 100% to 73.3%.
3. **A comparative evaluation of five heterogeneous architectures** under a unified transfer learning protocol, based on 10 independent runs (mean ± SD, 95% CI), reporting detection metrics (mAP@0.5, mAP@0.5:0.95, *Precision*, *Recall* and *F1-score*) and computational efficiency indicators (FPS, p50/p95 latency and GPU memory usage), with all architectures re-evaluated under a single framework-independent evaluator at harmonized decision thresholds so that the comparison is isoparametric at evaluation time.
4. **A custom dataset** of 1463 source images and 21,944 annotated instances across three classes (expanded to 3511 files and 52,664 instances through offline augmentation of the training subset) was captured on a university campus in Juliaca (Puno region, Peru), located at 3824 m a.s.l. The dataset represents a vehicle occupancy scenario under environmental conditions characteristic of the high-altitude Andes and was released with annotations.
5. **An integrated real-time monitoring system** that couples object detection (YOLOv8s), multi-object tracking (ByteTrack), a REST API backend (FastAPI), and a web-based visualization interface (Next.js 14) into a complete, deployable pipeline. Unlike prior comparative evaluations of parking detection models that report only offline benchmark metrics, this study validates the complete inference-to-display chain under real operating conditions by measuring the per-slot accuracy over 1395 observations across daytime and nighttime sessions. This validation also quantifies the throughput cost of the non-detection stages of the pipeline, which is decisive for architecture selection.

The study was organized around two main questions. The first is comparative and admits a single answer once the evaluation conditions are fixed: *which of the five architectures offers the most favorable accuracy–efficiency trade-off for real-time parking occupancy monitoring from a fixed camera under variable lighting?* The second is methodological and conditions the first: *to what extent does the dataset partitioning protocol determine the answer?* The two are not independent of one another. As shown below, the answer to the first question is stable only within a given protocol and reverses when the protocol changes; therefore, the comparative question cannot be settled without first settling the methodological question. Section 4.4 and Section 4.5 address the second question and, in doing so, establish the conditions under which the first question admits an answer.

## 2. Related Work

The literature on intelligent parking systems encompasses various approaches aimed at the automatic detection of available parking spaces and the analysis of vehicle occupancy using computer vision techniques. This review examines the main studies in the field, considering their methodological strategies, contributions, technical limitations, and their relationship with the current research.

### 2.1. Computer Vision-Based Intelligent Parking Systems

Computer-vision-based intelligent parking systems have gained increasing attention as an alternative to the automation of parking spaces monitoring using conventional surveillance cameras. Compared with solutions based on physical sensors installed in each parking slot, these approaches enable the simultaneous analysis of multiple spaces, reducing the modifications required for the existing infrastructure and facilitating scalability. However, their performance remains influenced by factors inherent to real-world environments, such as lighting variations, partial occlusions, weather conditions, camera viewpoints, and the visual complexity of urban settings.

Rafique et al. proposed a real-time intelligent parking management framework based on deep learning techniques, using YOLOv5s for vehicle detection on the PKLot dataset and real operational scenarios [6]. Their results showed that direct vehicle detection can be an efficient alternative to methods that focus exclusively on the individual classification of parking spaces. Similarly, Saputra et al. developed a YOLOv5-based system for the joint detection of vehicles and available parking spaces, demonstrating the applicability of one-stage object detectors to real-time vehicle monitoring tasks [15].

### 2.2. Deep Learning Models for Vehicle Detection

Advances in deep learning have reshaped the landscape of object detection in vehicle-related scenarios, particularly in systems where response speed is an operational constraint. Within this context, the (*You Only Look Once* YOLO) family has become one of the most widely used alternatives for real-time applications owing to its ability to maintain a balance between accuracy and inference speed. Recent studies have analyzed versions such as YOLOv8, YOLOv9, YOLOv10, and YOLOv11 in intelligent parking systems, incorporating strategies based on regions of interest and distributed inference schemes in edge/cloud architectures [7].

However, the predominance of one-stage detectors does not diminish the relevance of architectures based on different object detection approaches. Faster R-CNN with ResNet-50 FPN remains a benchmark among two-stage detectors because of its ability to localize objects with high accuracy in visually complex scenarios [12]. Its architecture combines a *Region Proposal Network* (RPN) to generate candidate regions with a subsequent stage for classification and bounding box refinement, achieving improved accuracy at the cost of greater computational demand.

From another perspective, the RT-DETR incorporates the transformer paradigm into real-time object detection [11]. Through an *end-to-end* approach, this model reduces the dependence on traditional procedures such as *Non-Maximum Suppression* (NMS), providing an alternative that seeks to balance computational efficiency and detection accuracy. These architectural differences justify the need for comparative evaluations that consider not only the predictive performance but also the implementation constraints inherent to the vehicle monitoring systems.

Within the YOLO family, YOLOv11 represents an incremental evolution from YOLOv8: C3k2 blocks replace C2f in the backbone, employing two internal bottleneck convolutions within a cross stage partial architecture to improve gradient reuse while keeping the parameter count of the small variant near 10 M; PSA modules in the neck provide lightweight multi-head attention for enhanced semantic aggregation without the cost of full self-attention [9]. YOLOv12 departs more substantially from the convolutional precedent by applying *area-attention*—self-attention within local rectangular partitions of the feature map rather than globally at the core of its architecture [10]. This design bounds the attention complexity to $O(n\;\cdot \;w_{a}^{2})$ rather than $O(n^{2})$, where $w_{a}$ is the fixed area width, enabling transformer-level contextual modeling at inference speeds that are competitive with small-scale CNN detectors. The R-ELAN backbone and optional FlashAttention support further reduce memory pressure during training. Critically, both characterizations rely on technical documentation and an arXiv preprint rather than peer-reviewed publications; where architectural details remain unconfirmed by independent reproduction, the comparisons in this study are grounded in empirical benchmark results rather than theoretical claims alone.

A complementary line of work addresses not the architectures themselves but the datasets on which they are benchmarked, and reaches a conclusion that converges with the central methodological argument of this study. Zunair et al. introduced RSUD20K, a road scene understanding dataset comprising over 20,000 high-resolution driving-perspective images from Bangladesh roads with 130,000 bounding box annotations across 13 object classes, motivated by the observation that object detectors trained on datasets from specific geographical locations struggle to generalize to different locations [16]. Their benchmark of state-of-the-art detectors—including YOLO variants and DETR-family models, and the same architectural paradigms compared here—shows that performance figures obtained on geographically homogeneous data overstate what a detector delivers when the scene distribution changes. RSUD20K addresses this problem through geographic and scene diversity at the dataset construction stage; the present study addresses its fixed-camera counterpart at the evaluation stage, showing that even within a single site, partitions that are not disjoint in acquisition time and camera viewpoint inflate performance estimates and can invert architecture rankings. The two approaches are complementary: diverse data quantify generalization across domains, whereas leakage-controlled partitioning quantifies it within one, and both expose the same failure mode of conventional random evaluation protocols.

Collectively, these architectures represent different contemporary paradigms of object detection. Modern YOLO models prioritize computational efficiency and real-time inference through one-stage detectors, whereas Faster R-CNN adopts a two-stage detector approach to achieve higher object localization accuracy. RT-DETR incorporates transformer-based mechanisms within an *end-to-end* framework, expanding the alternatives available for real-time detection. This architectural diversity highlights the need for comparative evaluations under homogeneous protocols, where the predictive performance and computational costs can be jointly analyzed for intelligent parking applications.

### 2.3. Comparative Analysis of Related Work

Table 1 summarizes the most representative studies reviewed, considering their objectives, employed architectures, main results, identified limitations, and their relationship with the proposed system.

The reviewed literature shows that real-time object detection models provide an effective alternative for intelligent parking systems by enabling the automated identification of vehicles and estimation of parking occupancy using computer vision techniques. Within this field, YOLO-based architectures have stood out for their ability to balance inference speed and accuracy, whereas Faster R-CNN remains a benchmark in scenarios in which localization accuracy is the primary concern. In contrast, RT-DETR incorporates a transformer-based paradigm and *end-to-end* detection, thereby expanding the range of options available for real-time applications.

Despite these advances, the reviewed systems still face limitations related to environmental variations, lighting changes, occlusions, and computational resource constraints. This highlights the need to compare architectures belonging to different detection paradigms under the same experimental conditions. Accordingly, this study analyzes one-stage CNN detectors, transformer-based models, and two-stage CNN detectors in a real university parking scenario located in the Peruvian Andean highlands, considering both detection accuracy metrics and factors associated with their real-time implementation.

## 3. Materials and Methods

### 3.1. Research Design

This study adopted a quantitative, experimental, and computational design to comparatively evaluate object detection architectures for intelligent real-time vehicle occupancy monitoring under variable lighting conditions. This study considered five models trained using a unified *transfer learning* protocol on a custom dataset collected on a university campus in Juliaca (Puno region, Peru), located at 3824 m above sea level (a.s.l). The evaluated models were YOLOv8s, YOLOv11s, and YOLOv12s, representing one-stage convolutional architectures; RT-DETR-L, based on *transformers*; and Faster R-CNN ResNet50-FPN, corresponding to the two-stage detection paradigm [13,14].

All models were initialized with weights pretrained on the COCO dataset and subsequently fine-tuned using the same training, validation, and test splits, along with a homogeneous set of hyperparameters. This procedure made it possible to control the experimental conditions and analyze the performance differences associated with the architectural characteristics of each model [17,18].

The evaluation included detection metrics (*Precision*, *Recall*, *F1-score*, *mAP@0.5*, and *mAP@0.5:0.95*), as well as computational efficiency indicators, including inference speed in FPS, latency at the p50/p95 percentiles, and GPU memory consumption. FPS values were obtained by considering only the inference stage with *batch* = 1, excluding additional operations from the complete processing pipeline. This consideration enabled more consistent comparisons with previous real-time detection studies [14,19].

Each experiment was performed ten times using fixed and sequential random seeds (42, 123, 456, 789, 1000, 1234, 2024, 2025, 3141, and 9999) to improve reproducibility and facilitate computational traceability. Because the detection metrics remain deterministic for a given random seed once the trained weights are established, the variability observed across runs arises primarily from the differences introduced during the training trajectories associated with each seed. The statistical uncertainty of the detection metrics was estimated through *bootstrap resampling* on the test set (B=1000), reporting 95% confidence intervals (95% CI). In contrast, computational efficiency metrics, particularly FPS and latency, were analyzed using the nonparametric Kruskal–Wallis test (α=0.05), followed by Dunn’s *post hoc* comparisons with Bonferroni correction.

The inference latency and FPS were measured using a single image with a resolution of 640×640 pixels and a randomly selected input tensor. Before each measurement, a warm-up stage consisting of 10 empty inferences was performed to stabilize the GPU memory and the cache behavior. Subsequently, the latency was calculated from 200 consecutive inferences by applying explicit GPU synchronization through torch.cuda.synchronize() before and after each execution to obtain more accurate timing measurements. This procedure was repeated for 10 independent runs, yielding a total of 2000 latency measurements per architecture. FPS was computed as $1000/\bar{{\mathrm{latency}}_{\mathrm{ms}}}$.

The research hypothesis established that differences exist in detection performance and, particularly, in computational efficiency among the evaluated architectures under homogeneous training conditions on the dataset collected from the analyzed campus.

It should be noted that RT-DETR-L (∼32 M parameters) has a larger architectural scale than the YOLOv8s, YOLOv11s, and YOLOv12s variants (∼11 M parameters). Therefore, comparisons within the YOLO family were interpreted as evaluations among models of comparable scales, whereas comparisons across paradigms were intended to characterize the trade-off between accuracy and computational efficiency under different architectural configurations, rather than as a strict comparison under equivalent parameter budgets.

### 3.2. General Methodological Workflow

**Figure 1:**
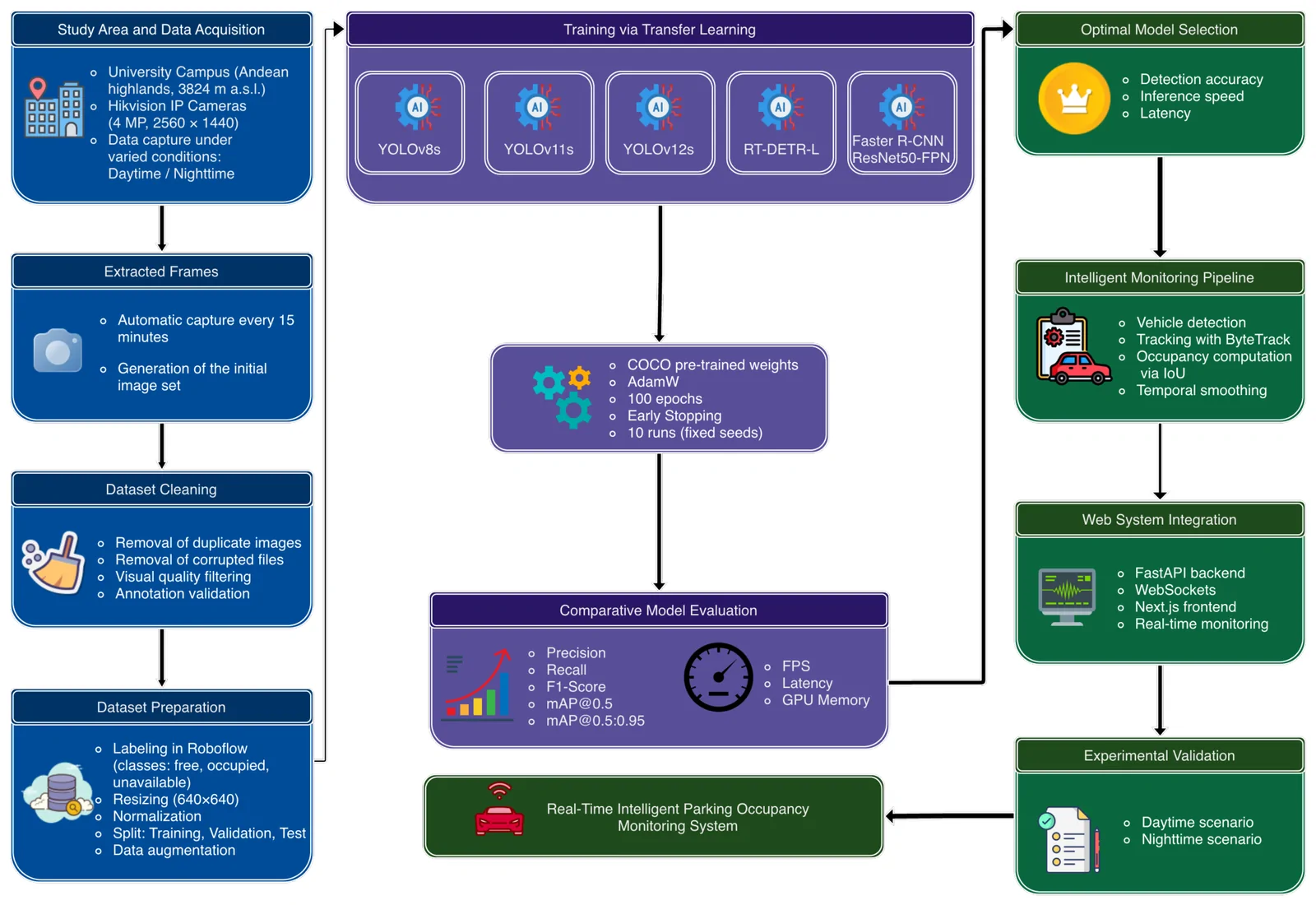
General workflow diagram of the proposed intelligent parking monitoring system methodology.

Figure 1 illustrates the overall workflow of the methodological process followed in this study, from dataset acquisition to the experimental validation of the proposed integrated system.

### 3.3. Study Area and Data Acquisition

Data acquisition was conducted in the parking areas of a university campus located in Juliaca (Puno region, Peru), at an approximate altitude of 3824 m a.s.l. The study site exhibits environmental conditions characteristic of the Andean highlands, including high solar radiation, significant light variation, and the presence of partial shadows. These characteristics pose challenges for computer vision systems designed for real-time vehicle monitoring [20,21].

Video acquisition was performed using a Hikvision DS-2CD1643G0-IZ IP camera, which was previously installed within the university campus. The device operated at a resolution of 2560×1440 pixels (4MP), with a 2.8–12 mm varifocal lens, H.264 encoding, and RTSP protocol streaming. During nighttime operation, the camera used EXIR infrared illumination with automatic switching between day and night modes. The camera was installed at an approximate height of 4–5 m above ground level, with an oblique viewing perspective and a field of view of approximately 70°, enabling the simultaneous capture of multiple parking slots while reducing the uncovered areas within the monitored region.

The acquisition process considered two main lighting scenarios: (i) daytime conditions with natural illumination between 06:00 and 18:00, and (ii) nighttime conditions with artificial illumination between 18:00 and 06:00. This distribution made it possible to incorporate the lighting variability present during the routine operation of the university parking facility into the dataset used in this study.

Frames were automatically extracted every 15 min from the original RTSP streams to reduce temporal redundancy while preserving spatial and environmental diversity within the dataset. The initial dataset consisted of 1463 images, distributed into training (1024; 70%), validation (293; 20%), and test (146; 10%) subsets. Subsequently, data augmentation techniques were applied exclusively to the training subset, increasing the number of images from 1024 to 3072 (augmentation factor: 3.0×). The final dataset comprised 3511 images in total.

To preserve the privacy of individuals and vehicles incidentally captured during data acquisition, vehicle license plates were anonymized using irreversible *hash* functions before they were permanently stored. Likewise, faces identified in the images were blurred using automatic Gaussian filters applied during the initial preprocessing stage of the dataset. These measures reduced the exposure of personally identifiable information and aligned data handling with the privacy and confidentiality principles established by current Peruvian personal data protection regulations.

### 3.4. Dataset Preparation

The frames extracted from the IP camera underwent a standardized preparation process to maintain spatial consistency, facilitate compatibility across models, and improve their robustness against environmental variations. All images were resized to a resolution of 640×640 pixels and normalized to the range [0,1], following the common configurations used in current real-time object detection systems [17,18].

The annotation process was performed using the Roboflow platform, employing polygon annotation tools that were adapted to the camera’s oblique perspective. Each parking slot was assigned to one of three categories: *free* (clearly available space), *occupied* (vehicle partially or fully visible within the parking slot), or *unavailable* (space whose status could not be determined owing to severe occlusions, the presence of traffic cones, temporary restrictions, or insufficient visibility). The annotations were subsequently exported in the YOLOv8 format, which is compatible with the architectures used in this study. Because this format uses rectangular bounding boxes, the polygon annotations were automatically converted to the minimum enclosing *bounding box* during the export process performed by the Roboflow.

The initial dataset consisted of 1463 images distributed into three subsets: training (1024 images; 70%), validation (293 images; 20%), and testing (146 images; 10). The partitioning was performed randomly using Roboflow while seeking to preserve the representation of both daytime and nighttime scenarios in each subset. However, because the images were captured using a fixed camera located in the same physical environment, this strategy did not establish a strict temporal separation between different acquisition days and sessions. Consequently, some subsets may contain visually similar scenes, particularly in situations where certain vehicles remain parked for extended periods.

To improve the generalization capability of the models and reduce the risk of overfitting to environmental variations, data augmentation techniques were applied exclusively to the training subset. The transformations included horizontal flipping, random rotation of $\pm 15^{\circ }$, brightness variation of up to ±25%, Gaussian blur of up to 1.5 pixels, and salt-and pepper noise applied to a maximum of 1.92% of the image pixels. These operations were intended to represent common visual conditions in the high-altitude Andean environment, such as intense illumination, partial shadows, reflections, and nighttime scenarios with reduced light. After this process, the training set increased to 3072 images, whereas the validation and test subsets remained unchanged.

It should be noted that, in addition to the offline data augmentation applied in Roboflow, the Ultralytics 8.4 framework applies its own set of training-time augmentation transformations (*online augmentation*) by default, which were not disabled during the experiments. The default values used were as follows: mosaic compositing probability =1.0; HSV hue shift $h_{gain}=0.015$; HSV saturation $s_{gain}=0.7$; HSV value $v_{gain}=0.4$; horizontal flip probability =0.5; translation fraction =0.1; scale gain =0.5; rotation =0.0; shear =0.0; perspective distortion =0.0; random erasing probability =0.4; mixup =0.0. These parameters correspond to the Ultralytics 8.4 defaults and were applied uniformly across all YOLO and RT-DETR-L runs (which used the same training interfaces). Faster R-CNN, implemented through Torchvision, did not use the Ultralytics pipeline and therefore was not subjected to this online augmentation layer. Consequently, the training images for YOLO and RT-DETR-L were subjected to a dual augmentation strategy (offline Roboflow transformations followed by the Ultralytics online pipeline), which should be considered when assessing strict protocol reproducibility and may have contributed to the high performance observed within the evaluated experimental conditions.

This strategy prevented the augmented images derived from the training set from being directly incorporated into the validation or test subsets of tha model. Nevertheless, because the initial partitioning was random rather than temporally disjoint, the presence of visual similarities between subsets cannot be completely excluded.

#### Dataset Curation Process

To improve the quality, consistency, and representativeness of the dataset, the corpus underwent systematic curation prior to the training stage. This procedure reduced visual noise, eliminated redundancies, and verified the spatial consistency of the annotations used as *ground truth*, following the methodological criteria reported in recent studies on intelligent parking detection [22,23].

The dataset curation process was structured into five sequential stages:

1. **Removal of duplicates and corrupted files:** Automated routines were applied to identify and discard duplicate, incomplete, or corrupted frames associated with temporary RTSP stream losses, H.264 compression errors, or transmission interruptions. This stage reduced the redundancy of static scenes and prevented the disproportionate representation of certain conditions in the training set.
2. **Temporal sampling and environmental balancing:** Frames were selected at 15-min capture intervals to increase the diversity of vehicle occupancy and reduce the temporal dependence between consecutive images. The distribution of daytime and nighttime scenarios was verified across the training, validation, and test subsets.
3. **Visual quality filtering:** Images with severe blur, overexposure, or extreme underexposure were discarded using automated metrics based on the variance of the Laplacian and intensity histogram analyses. This process helped reduce potential annotation errors resulting from degraded visual conditions.
4. **Geometric validation of annotations:** The polygon regions associated with parking slots were spatially verified to evaluate their correspondence with the actual occupancy areas defined in the Roboflow. The annotations were checked to ensure valid geometric boundaries and consistency across the frames with similar visual characteristics.
5. **Manual review of ambiguous cases:** Images containing partial occlusions, strong shadows, reflections, or external elements such as traffic cones and restricted areas were manually inspected by the annotators. Cases in which the occupancy status could not be determined with sufficient confidence were reclassified into *unavailable* categories.

In addition, the subset used to calculate the inter-annotator agreement was independently reviewed by a second evaluator with experience in image annotation for computer vision. This procedure enabled the early detection of semantic inconsistencies and improved the overall robustness of the annotation process before the final training of the evaluated architecture.

### 3.5. Model Training and Comparison

The training phase constituted the core experimental component of the research, which aimed to comparatively evaluate the performance of different object detection architectures under a controlled protocol that shared the same dataset splits, evaluation metrics, and hyperparameter targets. Framework-specific differences, including the Ultralytics online augmentation pipeline applied only to YOLO and RT-DETR-L, and the lower learning rate required by Faster R-CN, were explicitly documented to allow accurate interpretation of cross-framework comparisons. The study considered five models organized into two methodological groups: (i) a primary group consisting of YOLOv8s, YOLOv11s, and YOLOv12s, representing modern variants of the YOLO family designed for real-time detection applications [10,13] and (ii) a reference group composed of RT-DETR-L and Faster R-CNN ResNet50-FPN, used to contextualize the performance of the YOLO architectures against transformer-based paradigms and two-stage detectors [14].

All models were initialized with weights pretrained on the COCO dataset and subsequently fine-tuned using the university dataset. The experimental protocol maintained the same training, validation, and test splits for all five architectures, as well as equivalent training configurations, to control the comparison conditions across the obtained results.

#### 3.5.1. Transfer Learning Protocol

The unified training protocol applied to the five architectures was structured into four stages:

1. **Initialization with COCO weights:** Each model was initialized with weights pretrained on the COCO dataset, leveraging visual representations that had been previously learned from a large set of object categories and general detection scenarios [17,18].
2. **Fine-tuning on the custom dataset:** The models were fine-tuned using the augmented training subset of the university dataset to adapt the learned visual features to the specific domain of university parking facilities under varying environmental conditions.
3. **Homogeneous hyperparameters:** All architectures were trained using an input resolution of 640×640 pixels, a *batch size* of 16, an initial learning rate of 0.01 with *cosine annealing* decay to 0.0001, the AdamW optimizer, and a *weight decay* of 0.0005. Training was conducted for 100 epochs, incorporating *early stopping* with a patience of 20 epochs and monitoring *mAP@0.5* on the validation set.
4. **Computational infrastructure:** All experiments were conducted on a single NVIDIA RTX 3080 GPU with 10 GB of VRAM, using PyTorch 2.6 and CUDA 12.4. Each training process was repeated ten times using fixed random seeds (42, 123, 456, 789, 1000, 1234, 2024, 2025, 3141, and 9999) to evaluate the variability associated with the training process and improve experimental reproducibility.

The complete *hardware* and *software* environments used during the experiments are listed in Table S1 to ensure the reproducibility of the results.

#### 3.5.2. Model-Specific Considerations

YOLOv8s, YOLOv11s, and YOLOv12s were trained using the Ultralytics 8.4 library, which provides a unified interface for the training, validation, and inference stages of real-time object detection models. In the case of YOLOv12s, FlashAttention support was enabled when the available CUDA version allowed its use [10].

RT-DETR-L was also trained using the pipeline provided by Ultralytics 8.4, maintaining an experimental workflow comparable to that of the YOLO models. However, it should be considered that RT-DETR-L presents a larger architectural scale (∼32 M parameters) compared with the evaluated YOLO-s variants (∼11 M parameters). Therefore, comparisons between the two groups were interpreted as analyses between architectural paradigms with different complexity characteristics, rather than comparisons under equivalent parameter budgets.

Table 2 summarizes the configurations of the five architectures. Faster R-CNN was implemented using the fasterrcnn_resnet50_fpn_v2 function available in torchvision 0.21. The architecture employed a ResNet-50-FPN backbone pretrained on COCO, a standard *Region Proposal Network* (RPN), and the ROIAlign mechanism for multiscale feature extraction. The anchor configuration considered scales {32,64,128,256,512} pixels and aspect ratios {0.5,1.0,2.0}. During inference, the non-maximum suppression (NMS) threshold was set to an IoUof=0.5. The model was optimized using AdamW with a fixed learning rate of $1\times 10^{-4}$, *weight decay* of $5\times 10^{-4}$, and an additional *cosine annealing* decay scheme ($T_{max}=100$). Unlike YOLO and RT-DETR, which were trained with an initial learning rate of 0.01, Faster R-CNN used a lower learning rate ($1\times 10^{-4}$), following common configurations for fine-tuning two-stage detectors based on ResNet-FPN.

#### 3.5.3. Optimal Model Selection Criteria

The model selection was based on Pareto dominance in the (mAP@0.5:0.95, FPS) space, which requires no weighting. Under the random partitioning protocol, YOLOv8s simultaneously attains the highest accuracy and highest throughput and therefore dominates the remaining four architectures; thus, the Pareto front reduces to a single point. Section 4.5 repeats this analysis under the disjoint partition D3, where the front expands to four non-dominated architectures. The composite scoring function below was retained for secondary illustrative analysis:

(1) $$S=0.6\;\cdot \;mAP@0.5:0.95_{norm}+0.25\;\cdot \;FPS_{norm}+0.15\;\cdot \;\left(1-Lat_{p95,norm}\right)$$

where the *norm* subscripts represent min–max normalization across the complete set of evaluated architectures.

The weights were defined according to the operational requirements of the system: detection quality was considered the primary criterion (0.6), followed by processing speed (0.25) and inference latency (0.15). In addition, the minimum operational constraints of FPS ≥25 and p95 latency ≤40 ms were established to ensure real-time operation.

Table 3 summarizes the criteria, their weights and the operational constraints applied. The sensitivity of the final ranking was evaluated by testing eight alternative weighting configurations that maintained the priority ordering of detection accuracy over speed over latency, confirming the stability of the selected model under all evaluated combinations of weightings. The performance metrics considered during the comparison included *Precision*, *Recall*, *F1-score*, *mAP@0.5*, *mAP@0.5:0.95*, FPS, p50/p95 latency, and GPU memory consumption.

The weighting scheme is examined in Section 4.5 using accuracy figures from the disjoint partition, where the Pareto front is not degenerate and the choice of weights matters.

Two properties of this scoring function require explicit examination. First, FPS and p95 latency are not independent criteria: Spearman’s rank correlation between them across the five architectures is −1.0000, since one is a monotonic transformation of the other. Including both therefore double-counts the efficiency dimension, and the composite score is reported here with this caveat. Second, min–max normalization over only five models forces the best value to 1 and the worst to 0 by construction, which may exaggerate differences.

To verify that the selection does not depend on either the weights or the normalization, 10,000 weight vectors were sampled uniformly from the simplex and the procedure was repeated under min–max, ratio, and *z*-score normalizations. YOLOv8s was selected in 100% of the cases under all three schemes. This is consistent with the Pareto analysis, in which YOLOv8s was the only non-dominated point.

### 3.6. Detection and Tracking Pipeline

The model selected based on the evaluation results was integrated into a real-time vehicle monitoring pipeline designed for the detection, tracking, and occupancy estimation of parking slots using continuous video streams. The complete system operated at a target rate of 17 frames per second  FPS and incorporated sequential modules for detection, multi-object tracking, geometric occupancy analysis, and temporal smoothing.

#### 3.6.1. Detection and Tracking with ByteTrack

The annotation targets, model outputs, and tracker inputs are all parking-slot state bounding boxes: the model classifies each slot directly into *free*, *occupied*, or *unavailable* and does not detect vehicles as distinct entities. The resulting state detections were processed using ByteTrack, a multi-object tracking algorithm that associates both high- and low-confidence detections to maintain persistent identifiers (*trackID*) across consecutive frames [24]. Tracking serves to distinguish consecutive occupancy events within the same slot when the identifier associated with an occupied slot changes, the system registers a new parking event rather than a continuation of the previous one, rather than following the moving vehicles. Tracking identifiers are discarded for the detection of the *free* class, which is not a trackable entity. Unlike approaches that directly discard low confidence detections, ByteTrack leverages this information during the association process with existing trajectories, helping to reduce trajectory fragmentation and improve tracking continuity under partial occlusions.

The tracking module used the default parameters of the framework, which have been widely adopted in multi-object tracking applications. This configuration allowed the maintenance of active trajectories during brief temporary occlusions and reduced the spurious associations generated by isolated detections.

#### 3.6.2. Occupancy Estimation and Temporal Stabilization

The occupancy of each parking slot was determined using a geometric criterion based on the spatial relationship between the bounding boxes detected by the model and the polygons that represented each parking slot.

First, a coverage score is computed between each detected bounding box $B_{j}$ and parking slot polygon $P_{i}$, as expressed in Equation (2):

(2) $$S\left(B_{j},P_{i}\right)=\frac{|B_{j}\cap P_{i}|}{|P_{i}|}$$

where $|B_{j}\cap P_{i}|$ is the intersection area between the bounding box and polygon, and $|P_{i}|$ is the total area of the parking slot polygon. This metric quantifies the degree of spatial correspondence between a detection and a slot polygon, and is used to *associate* each detection with one of the predefined slots; it does not determine occupancy. The instantaneous state of a slot is the class predicted by the model for the detection assigned to it. A detection is associated with a slot when the coverage ratio reaches $\theta_{\mathrm{assoc}}$ or when its center falls inside the polygon.

Normalization by $|P_{i}|$ rather than by the union $|B_{j}\cup P_{i}|$ (IoU) or by the box area $|B_{j}|$ is a deliberate design choice motivated by the geometry of the scene. Under the oblique viewpoint of a fixed elevated camera, the axis-aligned bounding box of a correctly parked vehicle systematically exceeds its slot polygon: the box encloses the full projected height of the vehicle body, whereas the polygon delimits only the ground-plane area of the slot. Consequently, the IoU is depressed by the portion of the box that lies outside the polygon even for unambiguous, correctly parked vehicles, and normalizing by $|B_{j}|$ penalizes taller or closer vehicles whose boxes are larger for reasons unrelated to slot membership. Normalizing by $|P_{i}|$ answers the operationally relevant question of what fraction of the slot is covered by the detection—and is invariant to those box-size effects. Cases in which a single box overlaps more than one polygon are resolved by assigning the detection to the slot that maximizes $S(B_{j},P_{i})$, with the box-center criterion acting as a tie-breaking and fallback rule; the per-slot accuracy reported in Section *Experimental Setup* evaluates the complete association-plus-classification chain and is therefore the end-to-end check on this design. The robustness of the downstream decision to the exact threshold value—variations below 1.5% in per-slot accuracy across θ∈[0.40,0.50]—further indicates that the pipeline is not critically sensitive to the association score.

Next, an instantaneous occupancy state is assigned:

(3) $$o_{i}^{\left(t\right)}=\left\{\begin{array}{cc} 1, & \max_{j}S\left(B_{j},P_{i}\right)\geq \theta_{\mathrm{coverage}}\\ 0, & \mathrm{otherwise} \end{array}\right.$$

In Equation (3), $o_{i}^{\left(t\right)}$ is the state of parking slot *i* at time *t* (1 = occupied, 0 = free), and $\theta_{\mathrm{coverage}}$ is the minimum coverage threshold. This threshold was optimized through an exhaustive search on the validation set using values θ∈{0.30,0.35,…,0.70}, minimizing the per-slot classification error. The optimal value was $\theta_{\mathrm{coverage}}=0.45$, with variations of less than 1.5% in the per-slot accuracy within the interval [0.40,0.50].

To reduce transient fluctuations (occlusions, lighting changes, or isolated errors), a temporal stabilization mechanism based on a sliding window of W=25 frames (approximately 1.5 s at 17 FPS) was incorporated. The stabilized state ${\hat{o}}_{i}^{\left(t\right)}$ is updated using hysteresis as follows:

(4) $${\hat{o}}_{i}^{\left(t\right)}=\left\{\begin{array}{cc} 1, & \mathrm{if}\;\frac{1}{W}\sum_{k=0}^{W-1}o_{i}^{\left(t-k\right)}\geq \theta_{on}\\ 0, & \mathrm{if}\;\frac{1}{W}\sum_{k=0}^{W-1}o_{i}^{\left(t-k\right)}<\theta_{off}\\ {\hat{o}}_{i}^{\left(t-1\right)}, & \mathrm{otherwise} \end{array}\right.$$

with asymmetric thresholds: $\theta_{on}=0.50$ (at least 13 of the 25 frames in the window detected as occupied, since 13/25=0.52≥0.50, whereas 12/25=0.48 does not satisfy the condition) to transition to the *occupied* state; returning to the *free* state requires the occupied fraction to drop below 0.10 (at most 2 of the 25 most recent frames detected as occupied, since 2/25=0.08<0.10; equivalently, 23 or more frames must show no occupation). In the notation of Equation (4), $\theta_{off}=0.10$.

It is important to distinguish between the two threshold levels: $\theta_{\mathrm{coverage}}$ (0.45) operates on the spatial coverage within a single frame, whereas $\theta_{on}$ and $\theta_{off}$ operate on the temporal fraction of the occupied frames within window *W*. The asymmetric design ($\theta_{on}=0.50>\theta_{off}=0.10$) creates a hysteresis band for values between 0.10 and 0.50: once occupied, the slot remains occupied as long as the occupied fraction stays at or above 0.10; once free, it transitions to occupied only when the fraction rises to 0.50 or above. This prevents rapid oscillation under marginal or transitional occupancy conditions.

Geometric operations were implemented using Shapely and NumPy, which enabled efficient real-time polygon intersection calculations.

### 3.7. Web System

The monitoring system was complemented by a web platform designed for the real-time visualization and management of vehicle occupancy within the university campus. The implemented architecture followed a distributed *full-stack* approach, integrating inference, data communication, and visualization components through an interface accessible from modern web browsers [19,21].

The backend was developed using **FastAPI**, which provides REST services for querying historical information and bidirectional communication through WebSockets for transmitting real-time occupancy events. This architecture enabled the integration of the detection and tracking pipeline with the visualization services while maintaining continuous updates of the system status.

The frontend was implemented using **Next.js 14** and TypeScript, employing an interactive interface to dynamically represent the status of each parking slot in real time. The application considered three operational states for each parking slot: *free*, *occupied*, and *unavailable*. It also incorporated modules for historical queries, occupancy statistics, and the automatic generation of downloadable reports for the operational analysis of university parking facilities.

Communication between the components was performed through data exchange in the JSON format, maintaining the separation between the inference and visualization modules. The system was deployed using Docker containers on a local institutional server to, facilitate dependency management and the reproducibility of the execution environment.

**Figure 2:**
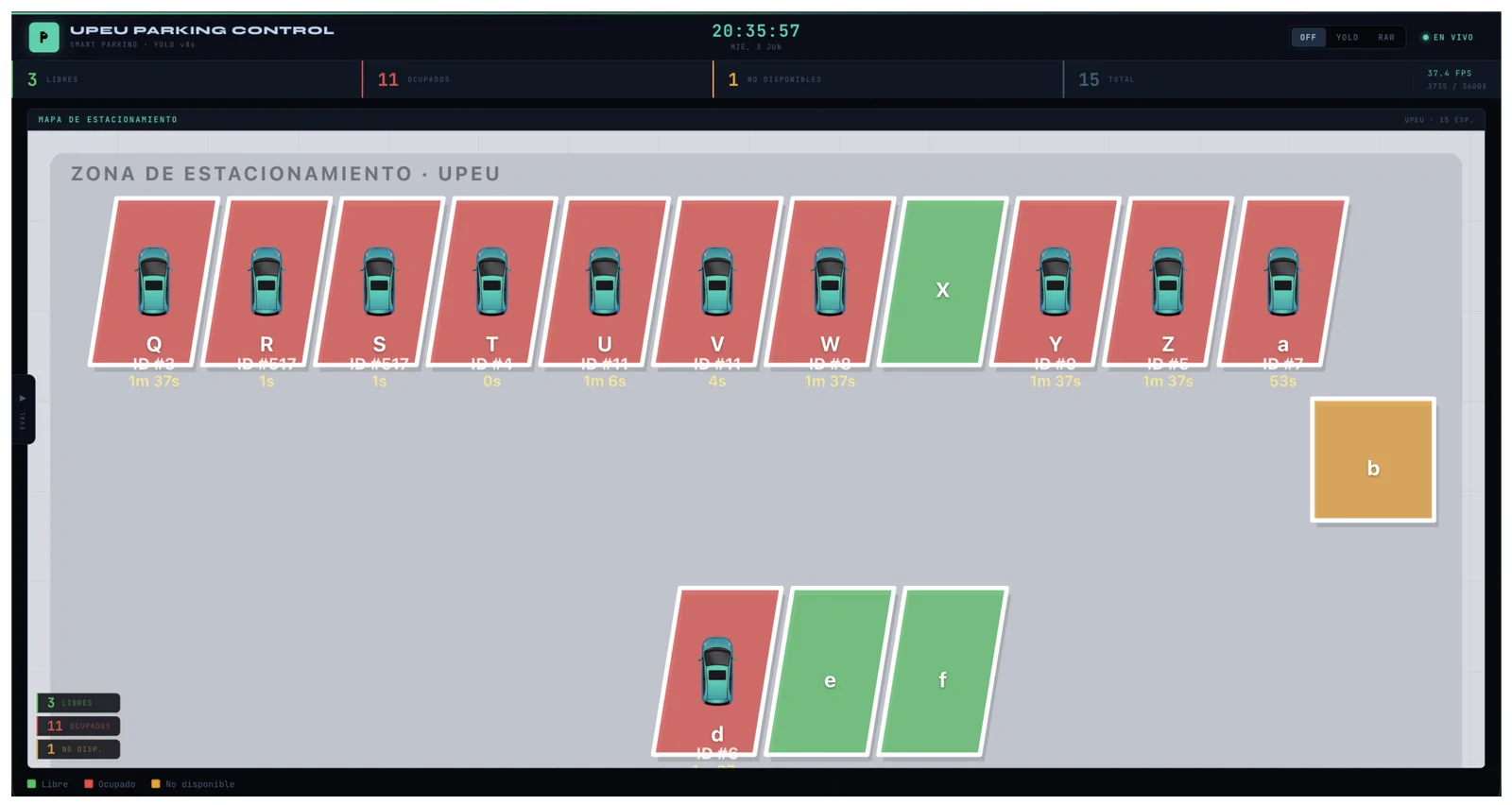
Web interface of the intelligent parking monitoring system. The real-time status of each parking slot (*free*, *occupied*, *unavailable*), aggregated occupancy statistics, and the operational history panel are displayed. The letters shown inside each rectangle (Q–Z, a–f) are the identifiers assigned to the individual parking slots in the monitored zone. interface of the intelligent parking monitoring system. The real-time status of each parking slot (*free*, *occupied*, *unavailable*), aggregated occupancy statistics, and the operational history panel are displayed.

Figure 2 illustrates the system interface during an active monitoring session, showing the real-time status of each parking slot along with the aggregated occupancy statistics.

Finally, the web system was integrated with the real-time inference pipeline, enabling continuous updates of the occupancy states during the experimental validation sessions, with a target operating rate of 17 FPS.

### 3.8. Experimental Validation

Field validation sessions were conducted on dates subsequent to and disjoint from the data collection period used for training (5–25 May 2026). No frame or session employed in field validation appeared in the training, validation or test subsets under any of the partitions evaluated; therefore, the reported slot accuracy constituted an out-of-sample estimate.

The experimental validation of the integrated system was conducted under real operational conditions within the university campus, considering two representative lighting scenarios: (i) a daytime scenario with natural illumination and (ii) a nighttime scenario with artificial light. These scenarios were selected based on their operational relevance and challenges documented in the literature regarding the performance of intelligent parking systems under varying lighting conditions [20,21].

The daytime evaluation was carried out through two independent sessions, whereas the nighttime evaluation consisted of a single session. Each session lasted 31 min and was conducted on different days, incorporating natural variations in vehicle density, ambient lighting conditions, and the presence of partial occlusions. During all sessions, the system operated continuously on real-time RTSP streams using the complete detection, tracking, and geometric occupancy estimation pipeline.

The metrics considered during validation included the detection performance, computational efficiency, and operational behavior of the integrated system. Table S2 summarizes the metrics used for the experimental evaluation.

The detection metrics were computed for the test subset using deterministic inference with fixed random seeds. The uncertainty associated with *mAP@0.5* and *mAP@0.5:0.95* was estimated through *bootstrap resampling* with B=1000 iterations, and 95% confidence intervals (CIs) were reported.

Computational efficiency metrics, particularly inference FPS, exhibited variability across runs owing to fluctuations inherent to the computational environment and the GPU workload. Therefore, differences among the architectures were analyzed using the nonparametric Kruskal–Wallis test with a significance level of α=0.05, followed by Dunn’s *post hoc* comparisons with Bonferroni correction.

A distinction was also established between the FPS obtained during standalone inference and those corresponding to an integrated system for the same test. The values used for the comparison among architectures correspond to standalone inference with *batch* = 1, whereas the operational performance of the complete system incorporates the additional costs associated with ByteTrack, geometric occupancy processing, and WebSocket-based data transmission.

Finally, the experimental results were compared with recent studies on intelligent parking detection reported in the literature [17,18,25], with the aim of placing the performance of the evaluated architectures within the context of real-world applications under the variable environmental conditions of the Peruvian highlands.

### 3.9. Ethical Considerations

This study was conducted in accordance with the ethical principles applicable to the processing of visual data acquired through institutional video surveillance systems. This study followed the provisions established by Peru’s Personal Data Protection Law No. 29733 and its associated guidelines for responsible handling of sensitive information.

The experimental protocol was reviewed and approved by the institutional ethics committee of a university located in the Peruvian Andean highlands before the start of the data acquisition and processing activities. Access to the video streams used in this study was restricted exclusively to the authorized research team.

To protect the privacy of individuals and vehicles incidentally captured in the images, the final dataset used for training and validation only stored anonymized information related to vehicle occupancy, tracking identifiers, and timestamps. No facial recognition, driver identification, or storage of directly identifiable personal data was performed at any stage of this study.

## 4. Results

### 4.1. Dataset Composition

The final dataset consisted of 1463 original frames captured in a university parking lot located in Juliaca (Puno region), Peru, at 3824 m  above sea level, under day and night lighting conditions. The corpus was partitioned into training (1024 images), validation (293 images) and test (146 images) subsets, following a 70/20/10 split. Data augmentation techniques were applied exclusively to the training subset using the Roboflow platform, which increased the number of images from 1024 to 3072. The final corpus used for training and evaluation of the models comprised 3511 images.

A total of 52,664 annotated instances were distributed across three classes: *free* (30,520 instances, 57.95%), *occupied* (18,634 instances, 35.38%) and *unavailable* (3510 instances, 6.66%). Table 4 summarizes the dataset split and Table S3 presents the class distribution.

#### Partitioning Protocol

Because the corpus consists of frames captured by a fixed camera, a random partition may place visually similar scenes into different subsets. To quantify this effect, five leakage-controlled partitions were constructed at the *source image* level, such that augmented copies of a given scene were never separated across subsets. Together with the random control R0, these constitute the six partitioning schemes analyzed throughout this study (R0, T2, T1, D1, D2 and D3).

- **R0 (control).** Random assignment, reproducing the original protocol under the revised pipeline. This establishes that any observed change is attributable to the partition and not the training environment.
- **T2 (day-stratified).** Complete, disjoint acquisition days were assigned so that the occupancy rate was balanced across the subsets (38.7%, 38.6% and 38.8%). This isolates the effect of removing the temporal leakage.
- **T1 (chronological).** The earliest 11 days of training and the latest six tests reproduced the deployment scenario.
- **D1 (cross-illumination).** The model was trained on 915 daytime images and evaluated using 378 nighttime images.
- **D2 (cross-viewpoint).** Training on one geometric template and evaluation on a second. The two templates share no polygon centroid and differ by a median displacement of 13.8 px at a 640 px resolution, while spanning the same range of acquisition days; thus, the contrast isolates the coordinate memorization from temporal drift.
- **D3 (cross-viewpoint and chronological).** Both constraints applied jointly.

Separating T1 from T2 is necessary because the acquisition period contains a regime shift: the mean occupancy is 51.5% between May 5 and May 15 and approximately 8% between May 16 and May 25. Therefore, a purely chronological split confounds leakage removal with distribution shift, and reporting both partitions allows the two effects to be distinguished (Table 5).

Each partition was audited for shared source images, shared acquisition days, minimum temporal separation between subsets. A complete verification report is provided in Supplementary Materials Table S1.

The instance counts reported above correspond to the augmented corpus. Because offline augmentation was applied exclusively to the training subset, file- and source-level counts differed by a factor of 2.40, both of which are reported in Table 6 to avoid ambiguity. No source image appeared in more than one subset of the dataset.

**Figure 3:**
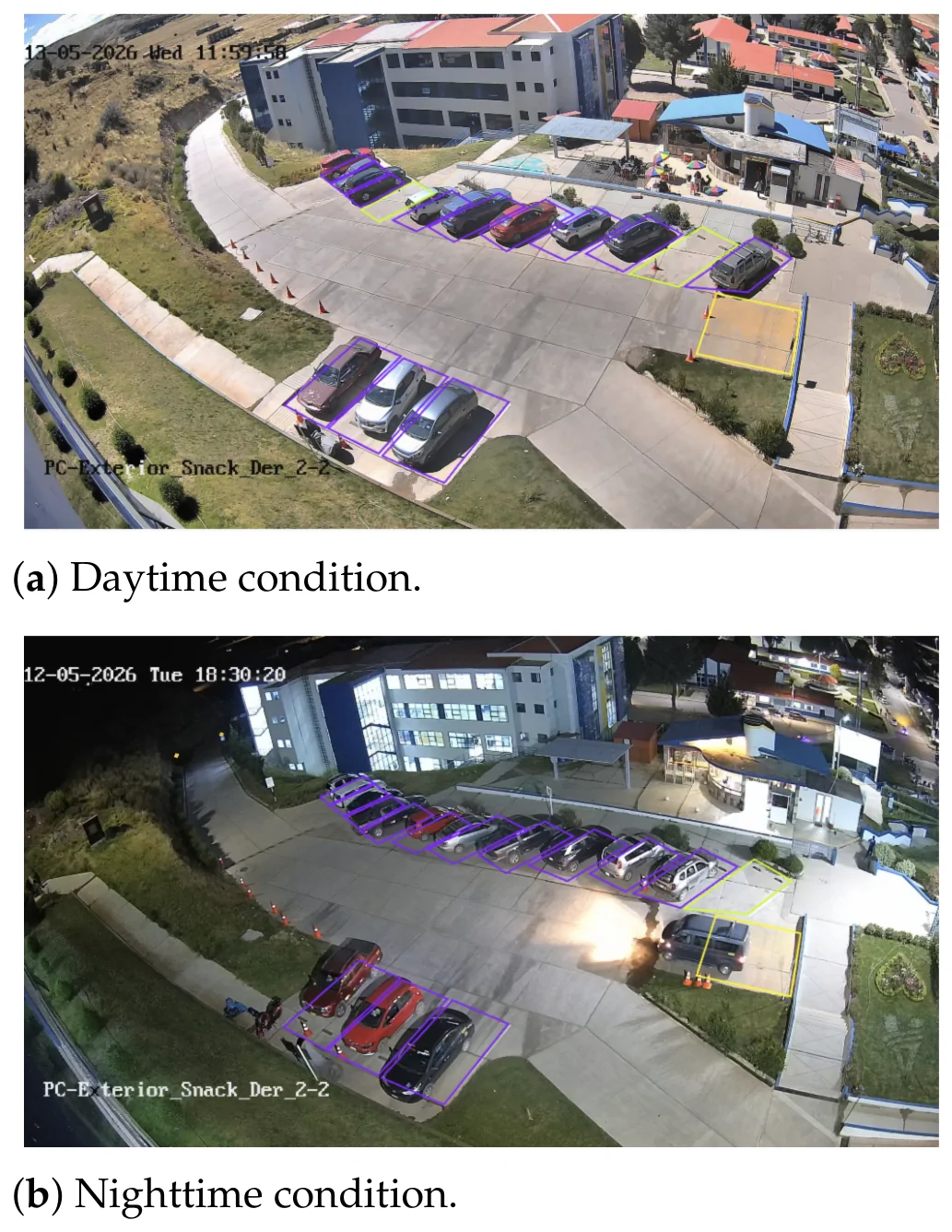
Representative samples of the dataset used in this study under different lighting conditions: (**a**) daytime scene and (**b**) nighttime scene.

Figure 3 shows representative samples from the dataset under both lighting conditions.

### 4.2. Training Convergence

**Figure 4:**
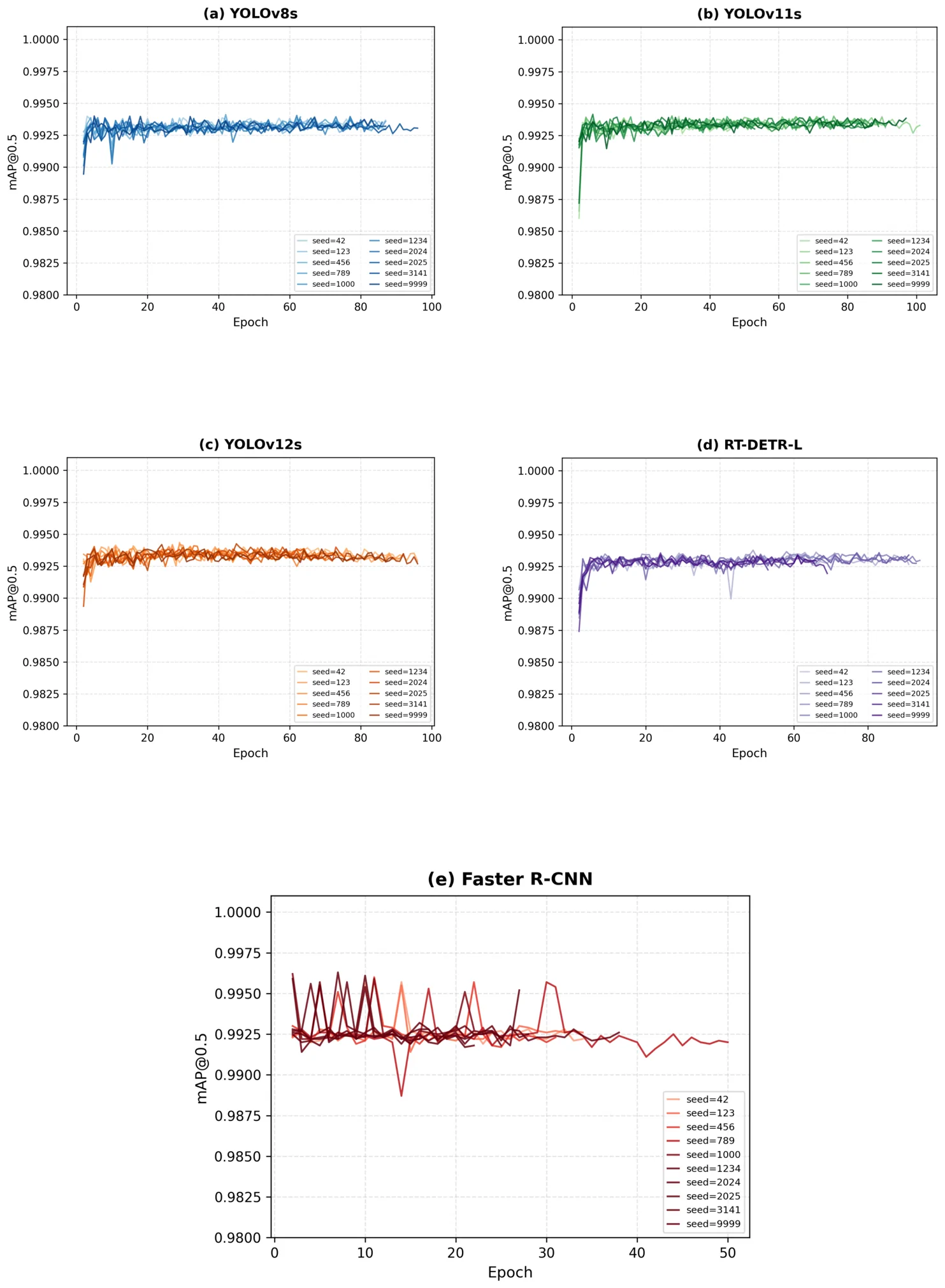
Convergence curves of mAP@0.5 on the validation set for the ten independent runs per architecture. Except for the single degenerate RT-DETR-L run (seed 42), the remaining runs show narrow convergence bands consistent with stable training and no clear evidence of overfitting under the evaluated conditions.

The five architectures were trained for a maximum of 100 epochs in 10 independent runs using reproducible fixed seeds (42, 123, 456, 789, 1000, 1234, 2024, 2025, 3141, and 9999). The *early stopping* criterion with a patience of 20 epochs on validation mAP@0.5 was triggered in most runs, indicating the progressive saturation of performance at intermediate stages of training and confirming that the 100-epoch limit did not constitute an active constraint on model convergence. Figure 4 shows the convergence curves of mAP@0.5 on the validation set for all runs per architecture. With the exception of the single degenerate RT-DETR-L run (seed 42), all remaining runs achieved stable convergence with no clear signs of overfitting within the evaluated protocol, as evidenced by the narrow band of curves across independent runs.

Table S4 summarizes the average epoch of *early stopping* activation and the final validation mAP@0.5 for each architecture over 10 independent runs. The stopping criterion was triggered in 49 of the 50 runs (98%), confirming that the 100-epoch limit did not constrain convergence. The YOLO architectures converged on average between epochs 80 and 83, whereas Faster R-CNN did so considerably earlier (epoch 30.1±8.1), consistent with its two-stage training scheme. For RT-DETR-L, one of the ten runs (seed 42) triggered the stopping criterion prematurely at the first epoch and was excluded from the average epoch computation; the remaining nine runs converged stably at 77.0±14.1 epochs. The degenerate outcome of seed 42 is attributed to numerical instability during transformer encoder initialization: under this particular seed, the model produced a validation mAP@0.5 of 0.0000 at epoch 1, and the Ultralytics framework terminated training before the full patience period elapsed, consistent with its handling of non-finite or zero-valued metrics in early epochs. This behavior is consistent with the known sensitivity of transformer-based architectures to weight initialization when fine-tuned on small custom datasets, where cross-attention layers may fail to attend to relevant feature regions under certain random states [11]. Despite the early termination, the mAP@0.5 and efficiency metrics of seed 42 were retained in the global statistics of Table 7; only the convergence-epoch average was computed over the nine non-degenerate runs.

### 4.3. Comparative Detection Performance

#### 4.3.1. Global Metrics

Table 7 presents the overall detection performance of the five evaluated architectures, expressed as the mean ± standard deviation and 95% confidence interval over 10 independent runs.

#### 4.3.2. Per-Class Performance

Table S5 presents the detection performance of the five evaluated architectures, disaggregated by class. In terms of mAP@0.5, the YOLO family of models showed high consistency across classes, with values above 0.994 for all model–class combinations. Faster R-CNN presented the lowest mAP@0.5 in the *free* (0.9899±0.0002) and *occupied* (0.9905±0.0030) classes, although it achieved mAP@0.5 =1.0000 in *unavailable*, being the only model to achieve perfect detection in that class. Regarding *Precision*, Faster R-CNN exhibited the highest variability among the architectures, particularly in the *occupied* class (0.9576±0.0342), suggesting instability in discriminating occupied spaces across independent runs. In contrast, all models achieved *Recall* ≥0.996 in the *unavailable* class, with YOLOv8s, YOLOv11s, YOLOv12s and RT-DETR-L obtaining perfect values (1.0000±0.0000).

**Figure 5:**
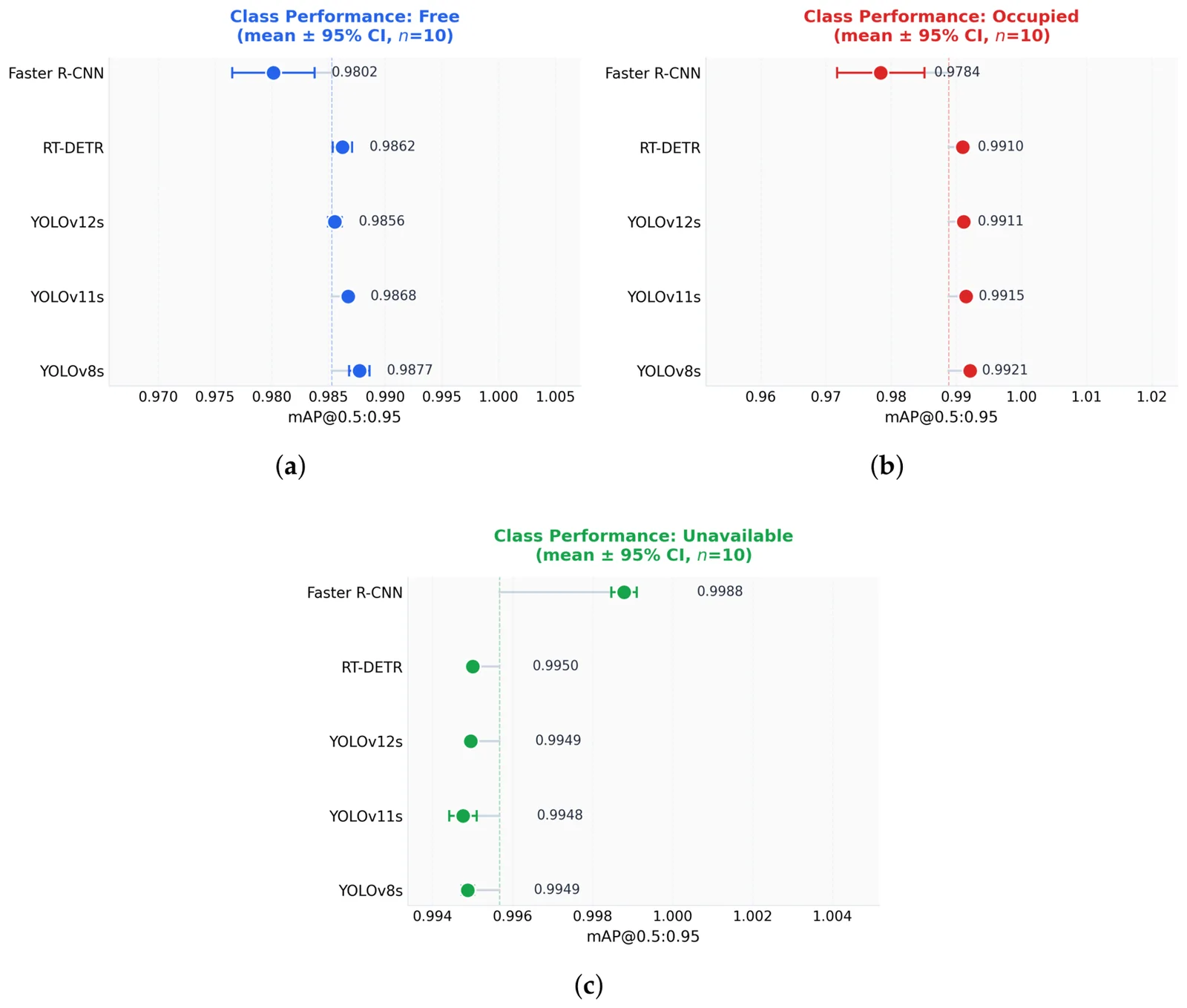
mAP@0.5:0.95 per class for the five evaluated architectures. (**a**) Class *free*. (**b**) Class *occupied*. (**c**) Class *unavailable*. The *unavailable* class achieved the highest performance across all evaluated models, while *free* presented the lowest values.

Figure 5 shows the mAP@0.5:0.95 per class for the five evaluated architectures. The *unavailable* class achieved the highest performance across all models, with Faster R-CNN obtaining the highest value (0.9988±0.0004) followed by RT-DETR-L (0.9950±0.0000). The *free* class presented the lowest values across all models, with YOLOv12s recording the minimum (0.9856±0.0009), whereas the *occupied* class showed an intermediate behavior. Faster R-CNN exhibited the highest inter-run variability in the *free* and *occupied* classes (SD=0.0051 and 0.0094 respectively), contrasting with the high stability observed in the YOLO family models.

**Figure 6:**
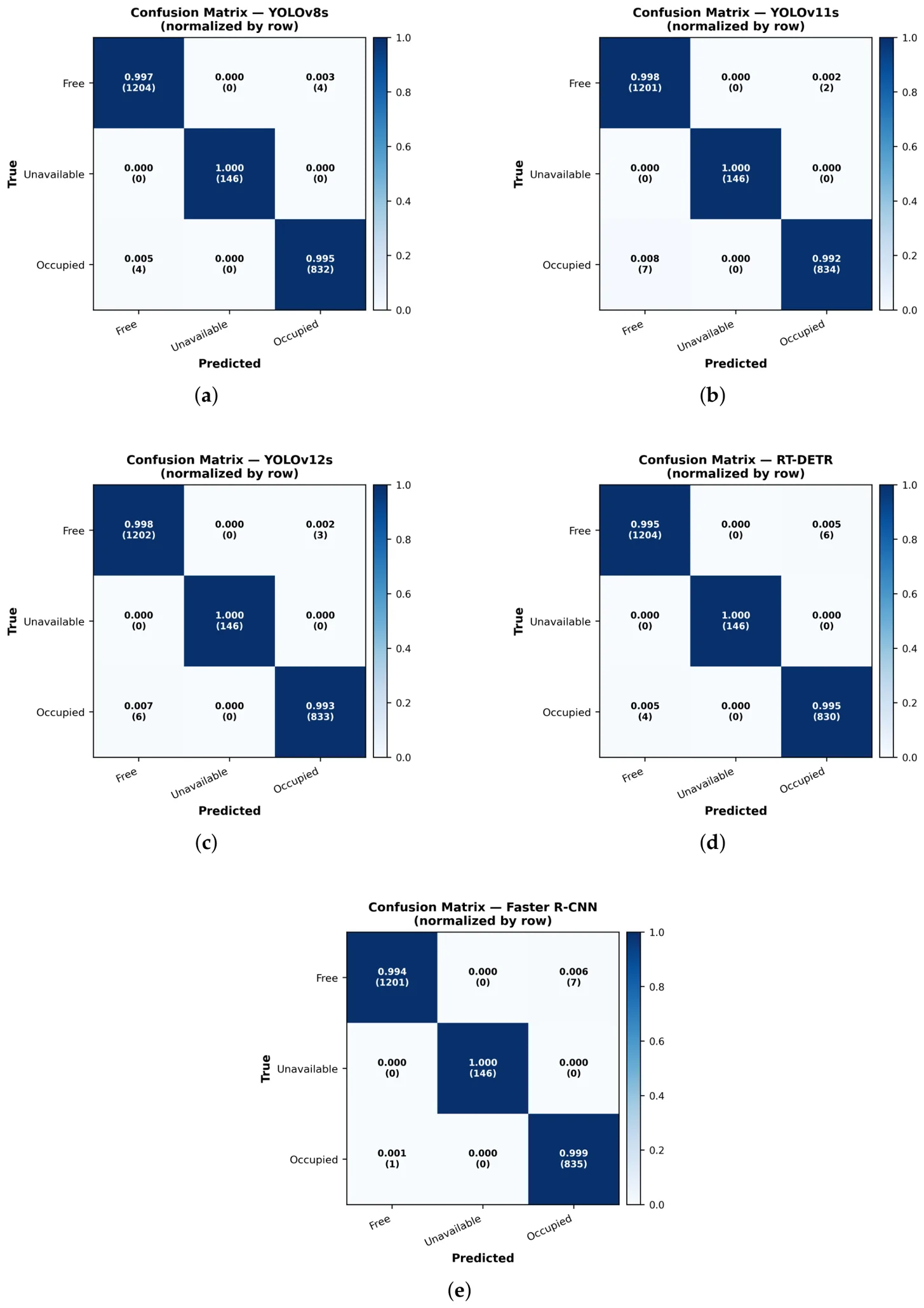
Normalized confusion matrices corresponding to the five evaluated architectures on the test set: (**a**) YOLOv8s, (**b**) YOLOv11s, (**c**) YOLOv12s, (**d**) RT-DETR-L and (**e**) Faster R-CNN. In all cases, a high concentration of observations is observed on the main diagonal, indicating a high discriminative capacity between the *free*, *unavailable* and *occupied* classes.

Figure 6 presents the normalized confusion matrices corresponding to the representa-tive run of each architecture, defined as the one whose mAP@0.5 was closest to the mean of the 10 independent runs, thus avoiding biases associated with selecting the best individual run.

#### 4.3.3. Computational Efficiency

Table 8 presents the computational efficiency metrics of the five evaluated architectures. YOLOv8s demonstrated the highest throughput (205.49 FPS [200.93,210.05]) and the lowest latency (p95: 5.24 ms [5.10,5.37]), satisfying both operational thresholds of ≥25 FPS and ≤40 ms. YOLOv11s achieved the second highest throughput (161.20 FPS [157.09,165.31]), also within operational limits. The YOLOv12s exhibited high variability in computational metrics across runs (${\mathrm{SD}}_{\mathrm{FPS}}=26.85$, ${\mathrm{SD}}_{\mathrm{GPU}}=1609$ MB), likely attributable to variable GPU load conditions during evaluation on the training server. RT-DETR-L presented the fourth highest throughput (41.07 FPS [40.63,41.51]), remaining above the ≥25 FPS threshold but with substantially higher latency (p95: 25.53 ms [24.78,26.28]). Faster R-CNN exhibited the lowest throughput among the evaluated models (26.97 FPS [26.42,27.52]), satisfying the minimum operational threshold with the highest p95 latency (37.91 ms [36.85,38.97]) and significantly lower GPU memory consumption (2968±4 MB) compared to the rest of the evaluated architectures, which is attributable to its torchvision implementation with static memory allocation during inference.

The Kruskal–Wallis test revealed statistically significant differences in FPS among the five evaluated architectures (H=47.06, p<0.001, $\varepsilon^{2}=0.96$). Dunn’s *post hoc* analysis with Bonferroni correction identified significant differences between YOLOv8s and the lower-throughput models (YOLOv12s, RT-DETR-L and Faster R-CNN), between YOLOv11s and the slower models (RT-DETR-L and Faster R-CNN), and between YOLOv12s and Faster R-CNN. No significant differences were observed between YOLOv8s and YOLOv11s, YOLOv12s and RT-DETR-L, or RT-DETR-L and Faster R-CNN (Table S6).

#### 4.3.4. Optimal Model Selection

The optimal model was selected using the composite scoring function defined in Equation (1), which balances the detection accuracy and computational efficiency, where the *norm* subscripts denote min–max normalization over the set of evaluated models.

Table 9 reports the composite score of the five architectures. YOLOv8s achieved the highest value (S=1.0000), satisfying both operational thresholds (FPS≥25 and p95 latency ≤40 ms) and ranking first among all evaluated architectures. This outcome is invariant to the weighting scheme: the Monte Carlo analysis of Section 3.5.3 returns YOLOv8s in 100% of 10,000 randomly sampled weight vectors under three normalization schemes, and a complementary check over eight structured weighting configurations reproduces the same result (Table S7). As noted in Section 3.5.3, this invariance reflects the fact that under random partitioning YOLOv8s is the single non-dominated point, so the scoring function has nothing left to discriminate.

Because the architectures were originally evaluated with two different programs—the Ultralytics validator for the YOLO and RT-DETR families, and pycocotools for Faster R-CNN and with different decision thresholds, the comparison was repeated using a single framework-independent evaluator. Two threshold regimes were distinguished, both identical across models: a detection regime (score ≥0.001) for mAP, which requires the full precision–recall, curve, and an operating regime (score ≥0.25) for precision, recall and $F_{1}$. The results are shown in Table 10.

Under this protocol, the two architectures are statistically indistinguishable in terms of detection quality, with overlapping confidence intervals. The precision disadvantage previously attributed to Faster R-CNN (0.9742±0.0228) was therefore an artifact of the lower decision threshold combined with a different evaluator and not a property of the architecture. The selection of YOLOv8s was based entirely on computational efficiency.

### 4.4. Effect of the Partitioning Protocol

The two leading architectures were retrained under each of the partitions defined in Section 4.1, keeping every hyperparameter unchanged so that the partition was the only varying factor. The results are presented in Table 11.

Three observations were made in this study. First, the control partition reproduces the originally published figure (0.9913 against 0.9916±0.0003), confirming that the revised pipeline is equivalent to the original despite the change in the hardware and software environment.

Second, the two sources of leakage were not additive in nature. Table 12 isolates each component: temporal separation alone costs 0.0085 in mAP@0.5:0.95 and viewpoint separation alone costs 0.0176, yet their conjunction costs 0.1085, that is, 4.16 times the sum of the individual effects. Neither constraint alone reveals the extent to which the model depends on the acquisition conditions being held fixed.

The superadditivity factor follows directly from the values in Table 11. For YOLOv8s, the cost of each constraint applied in isolation is the difference between the control partition and the corresponding partition as follows:

(5) $$\Delta_{\mathrm{temporal}}={\mathrm{mAP}}_{R0}-{\mathrm{mAP}}_{T1}=0.9913-0.9828=0.0085$$

(6) $$\Delta_{\mathrm{geometric}}={\mathrm{mAP}}_{R0}-{\mathrm{mAP}}_{D2}=0.9913-0.9737=0.0176$$

Equations (5) and (6) provide the cost of each constraint. Under an additive model, applying both jointly would cost their sum, 0.0085+0.0176=0.0261. The observed cost in Equation (7) is considerably larger:

(7) $$\Delta_{\mathrm{joint}}={\mathrm{mAP}}_{R0}-{\mathrm{mAP}}_{D3}=0.9913-0.8828=0.1085$$

The interaction term and the superadditivity factor are therefore

(8) $$I=\Delta_{\mathrm{joint}}-\left(\Delta_{\mathrm{temporal}}+\Delta_{\mathrm{geometric}}\right)=0.1085-0.0261=0.0824,\;F=\frac{\Delta_{\mathrm{joint}}}{\Delta_{\mathrm{temporal}}+\Delta_{\mathrm{geometric}}}=\frac{0.1085}{0.0261}=4.16$$

The same computation for YOLOv11s gives $\Delta_{\mathrm{temporal}}=0.9910-0.9793=0.0117$ and $\Delta_{\mathrm{geometric}}=0.9910-0.9573=0.0337$, summing to 0.0454, against $\Delta_{\mathrm{joint}}=0.9910-0.8763=0.1147$, which yields I=0.0693 and F=2.53. Both architectures exhibit superadditivity of different magnitudes.

Two caveats apply. First, D2 and D3 differ not only in the additional chronological constraint but also in the size of their training subsets (539 against 491 source images); therefore, a small part of the joint effect may be attributable to the reduction in training data rather than to the interaction itself. Second, *F* is the ratio of differences between means and inherits their dispersion; given the between-seed standard deviations involved (±0.0073 for D2 and ±0.0202 for D3), it should be read as an order of magnitude—the joint effect is several times the sum of its parts—rather than as a precisely estimated quantity.

**Figure 7:**
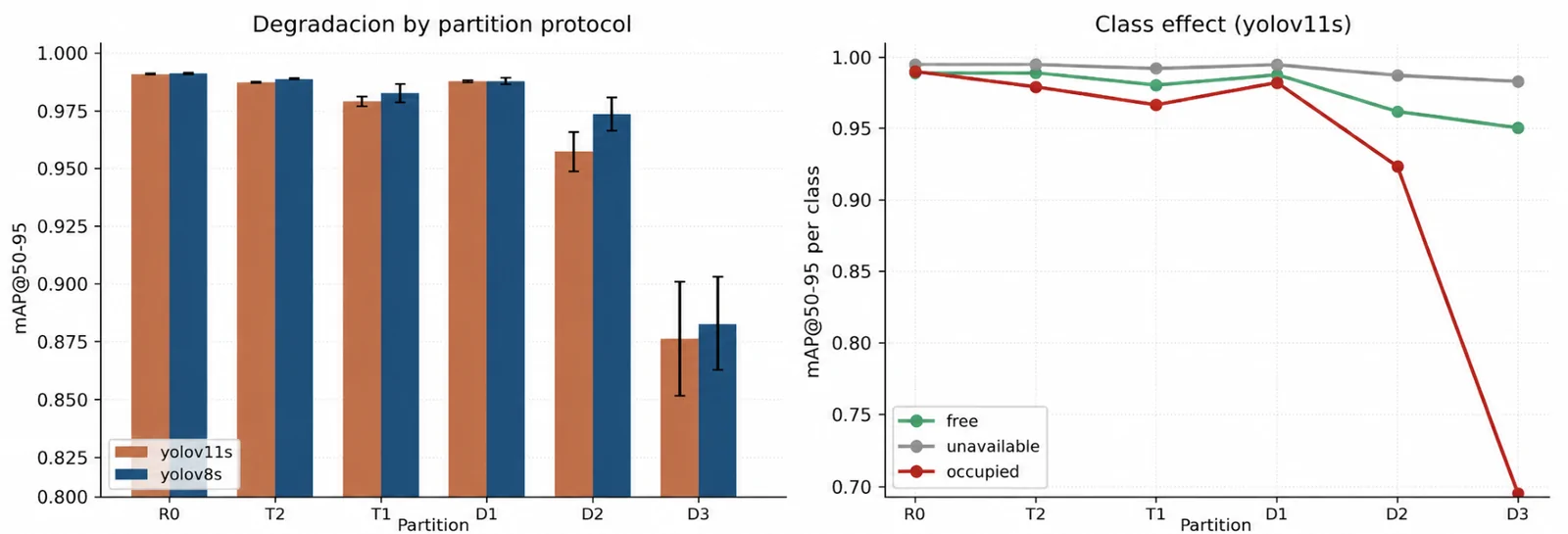
Detection performance under the six partitioning protocols (**left**) and per-class performance under the six partitioning protocols (**left**) and per-class decomposition of the effect (**right**). Error bars denote one standard deviation across seeds.

Third, degradation was concentrated in a single class. The *occupied* class falls from 0.9899 under the control partition to 0.7242 under D3, whereas an *unavailable*—a slot whose position and label never change—remains at 0.9876. The between-seed standard deviation also increased by a factor of 50, from ±0.0004 to ±0.0202, indicating that the original protocol concealed not only the level of performance but also its stability. Figure 7 shows the effects of this approach.

Regarding the comparison between architectures, YOLOv8s was significantly superior to YOLOv11s under D2 (Δ=0.0163, p<0.001, ten seeds), whereas under D3 the difference (Δ=0.0065) was not statistically distinguishable, as the between-seed variance grew with protocol difficulty. The consistent finding across all conditions was degradation relative to the control, whose effect size far exceeded the dispersion across seeds.

#### Experimental Setup

The integrated system, composed of YOLOv8s for vehicle detection, ByteTrack for tracking, FastAPI for real-time service management and Next.js 14 for web visualization, was validated under real operating conditions in the same parking lot used during dataset acquisition. The evaluation was conducted through three independent sessions carried out on different days: two daytime sessions and one nighttime session, each lasting 31 min.

**Figure 8:**
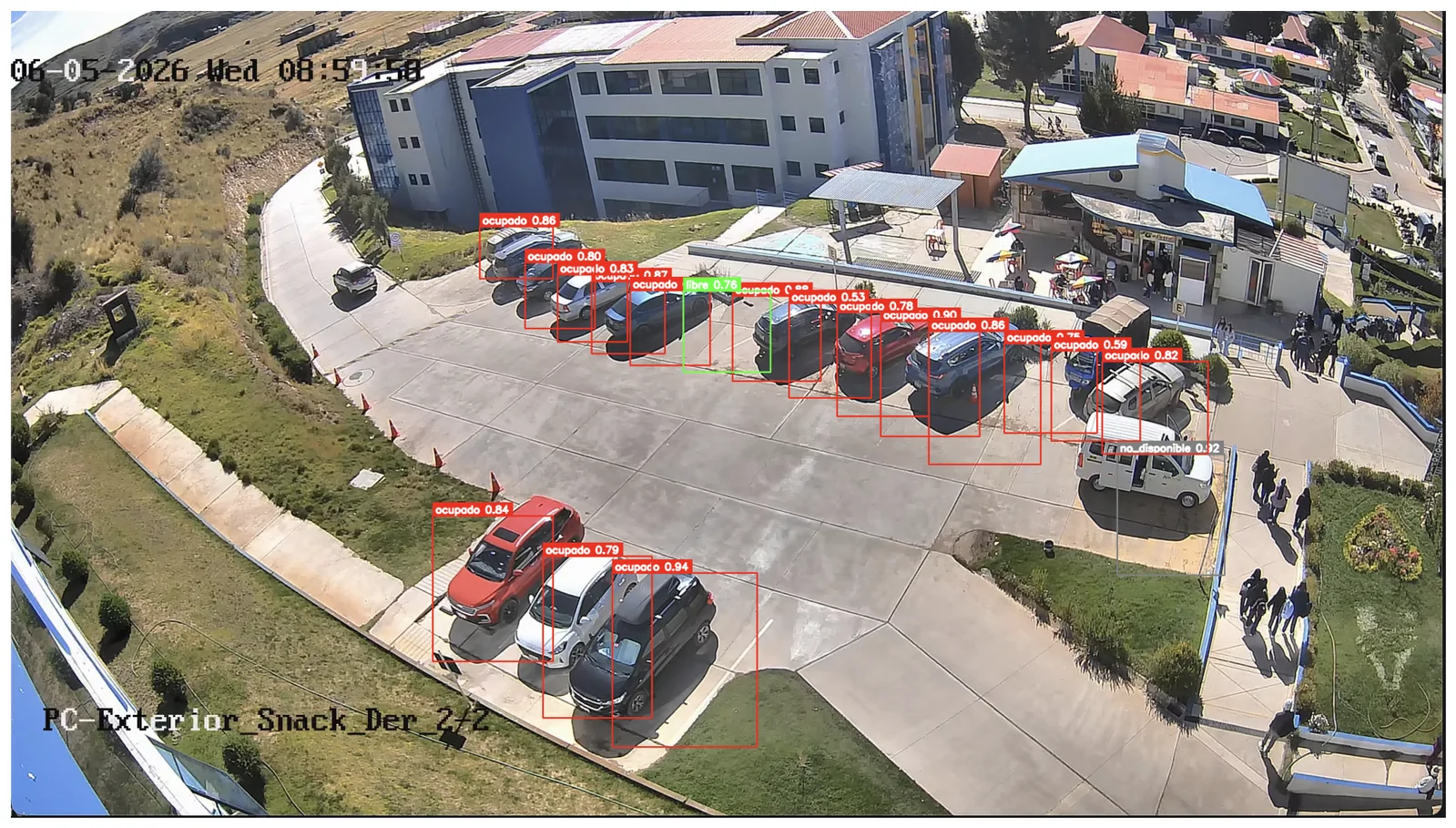
YOLOv8s detection system output on the real-time video stream during a daytime validation session. Bounding boxes with class labels and confidence scores are illustrated on the monitored parking slots. Red frames denote slots detected as *occupied* and green frames slots detected as *free*; the labels shown in the image are in Spanish, as displayed by the deployed interface.

Figure 8 shows a representative sample of the detection system output during the validation session, which illustrates the bounding boxes generated by YOLOv8s in the real-time video stream.

The ground truth was established through direct human observation of the video stream, recording the occupancy status of each parking slot at regular one-minute intervals. Subsequently, the manual records were compared with the predictions generated by the system to determine the occupancy detection performance. Each observation unit corresponds to a single parking slot at a single one-minute recording instant; therefore, the total observation count equals $N_{\mathrm{slots}}\times N_{\mathrm{timestamps}}$. With 15 monitored parking slots and 31 recording instants per session (one per minute over 31 min), each session generated 15×31=465 observations. Across two daytime sessions and one nighttime session, the complete validation comprised 3×465=1395 observations in total (930 during the daytime and 465  during the night).

The validation focused on three main metrics: *Slot Accuracy*, defined as the proportion of correctly classified slots; *False Positive Rate* (FPR), the percentage of free slots incorrectly classified as occupied; and *False Negative Rate* (FNR), the percentage of occupied slots incorrectly classified as free. For the purposes of FPR and FNR calculation, the task was treated as a binary free-vs.-occupied classification; slots whose ground-truth state was *unavailable* were excluded from these two rate calculations, as the unavailable category does not contribute to free or occupied reference counts. For each lighting condition, 95% confidence intervals were calculated using Wilson’s method.

Table 13 presents the validation metrics of the integrated system for both lighting conditions. The per-slot accuracy (*Slot Accuracy*) reached 87.5% [85.2,89.5] under daytime conditions and 91.8% [89.0,94.0] under nighttime conditions. Daytime sessions a showed lower FPR (1.6%) but a higher FNR (13.3%), indicating that detections were occasionally missed under natural lighting variability. Nighttime sessions presented a higher FPR (9.3%) but a lower FNR (7.9%), attributable to spurious activations under artificial lighting.

The confidence intervals for both metrics overlapped between conditions: slot accuracy was 87.5% [85.2,89.5] in the daytime sessions and 91.8% [89.0,94.0] in the nighttime session, while the false positive rate was 0.3–8.3% and 5.0–16.7%, respectively. Therefore, the apparent nighttime advantage is not statistically distinguishable at the current sample size and rests on a single 31-min session. This should be treated as preliminary and is not claimed to be a finding of this study.

### 4.5. Model Ranking Under the Disjoint Partition

The selection framework in Section 3.5.3 was built based on the accuracy figures obtained under random partitioning. Because the analysis in Section 4.4 identifies D3 as the more rigorous generalization estimate, the five architectures were retrained under D3 with ten seeds each to verify whether the ranking was preserved. It is not. Table 14 reports the accuracy, throughput, and resulting positions of each architecture under both protocols.

The ordering obtained under random partitioning is essentially the inverse of that obtained under disjoint partitioning. The architecture ranked last under R0-Faster R-CNN, which was the most accurate under D3, by a margin that was statistically significant against the second-placed model (Δ=0.025, p≈0.01) and large against YOLOv8s (Δ=0.050, p<0.001).

This behavior is consistent with the inductive biases of each species. A two-stage detector generates candidate regions dynamically through its region proposal network, whereas single-stage detectors with a fixed grid and predefined anchors can exploit a constant spatial configuration, an advantage that disappears when the camera viewpoint changes. The random partition rewards precisely the capacity that does not transfer.

**Figure 9:**
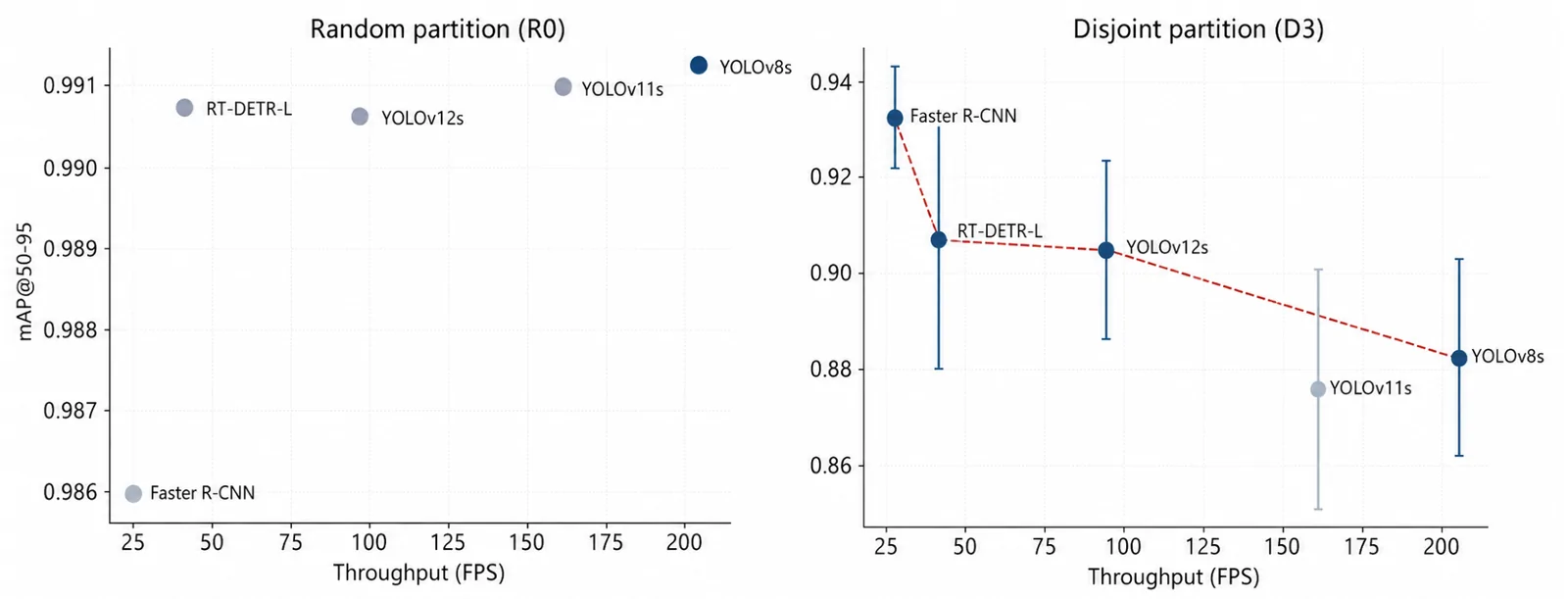
Accuracy –throughput space under random partitioning (**left**) and under the disjoint partition D3 (**right**). Non-dominated architectures are shown in dark blue and dominated architectures in grey; the Pareto front is shown as a dashed line. Under R0 the front reduces to a single point and the five architectures are indistinguishable in accuracy; under D3 a genuine trade-off emerges. Error bars denote one standard deviation across ten seeds.

Figure 9 contrasts the accuracy–throughput space under both protocols and makes the structural consequence visible: under random partitioning the Pareto front reduces to the single point occupied by YOLOv8s, whereas under the disjoint partition it expands to four non-dominated architectures spanning the accuracy–efficiency trade-off.

The consequences of the model selection are summarized in Table 15. Under R0 the Pareto front contains a single architecture; therefore, the composite score is redundant, and any weighting returns YOLOv8s. Under D3 four of the five architectures are non-dominated, and the selection becomes genuinely weight-dependent.

YOLOv8s remains the most frequently selected architecture under D3 because its throughput advantage between five and eight times that of Faster R-CNN outweighs the five mAP@0.5:0.95 points. Therefore, the recommendation of this study stands, but its basis changes: YOLOv8s is not the most accurate architecture; it is the fastest among the non-dominated architectures. Thus, the recommendation is explicitly conditional on the deployment profile rather than absolute. It holds for the operating regime targeted by the integrated system of this study—continuous video inference on a single commodity GPU, where the detector must leave most of the per-frame budget available for tracking, occupancy geometry, temporal smoothing and transmission (Section 8 quantifies this margin at the 17 FPS operating point). The 73.3% selection frequency should be read accordingly: it indicates that most, but not all, plausible weightings of accuracy and efficiency favor YOLOv8s, and the complementary 26.7%—concentrated in accuracy-dominant weightings—favors Faster R-CNN (20.4%) or YOLOv12s (6.3%). Deployments that prioritize accuracy over latency and can accommodate inference below 30 FPS are better served by Faster R-CNN, which is the most accurate architecture under the disjoint partition (mAP@0.5:0.95 =0.9325) by a statistically significant margin.

## 5. Discussion

The results show that the five evaluated architectures achieved high mAP@0.5 values on the test set. However, these results must be interpreted within the context of the experimental protocols. Because the images were captured with a fixed camera in the same environment and randomly split by Roboflow, there was likely to be visual similarity among the subsets, especially in scenes where vehicles remained parked for extended periods. Consequently, the high mAP values reflect the performance under these conditions but do not constitute sufficient evidence of generalization ability across completely independent days, sessions, or parking lots.

A more in-depth analysis of the mAP@0.5 distribution is required. The absolute difference between the highest-performing model (YOLOv8s: 0.9948) and the lowest (Faster R-CNN: 0.9935) was only 0.0013, which is less than 0.2 percentage points, across architecturally heterogeneous models spanning one-stage CNNs, a transformer-based end-to-end detector, and a two-stage detector with approximately four times more parameters. This near uniform convergence is unlikely to reflect equivalent true detection capability across all paradigms; rather, it is consistent with a ceiling effect in which the task, as defined, did not present a sufficient visual challenge to differentiate the models’ representational power. Three characteristics of the experimental setup may have contributed to this result: (i) the fixed camera provided a constant viewpoint and a constrained set of spatial configurations for each parking slot, making the detection problem geometrically predictable once the model had seen several examples from that perspective; (ii) the three-class taxonomy included an **unavailable** category whose visual cues (traffic cones, marked areas, and severe occlusions) were distinctive enough to be nearly perfectly detected by all architectures, which inflated the macro-average mAP; and (iii) the dataset originated from a single physical environment, limiting the diversity of background textures, vehicle types, and lighting configurations that might otherwise differentiate architectures with different inductive biases. These observations do not diminish the validity of the comparative efficiency analysis, where the Kruskal–Wallis test identified substantial and significant differences (H=47.06, p<0.001). However, they imply that model selection based solely on mAP@0.5 under this protocol would be uninformative. The partitioning analysis in Section 4.4 confirms this reading: under the fully disjoint partition D3, the spread between the most and least accurate architectures widens from 0.0013 to 0.0562 in mAP@0.5:0.95, and the Pareto front expands from a single point to four non-dominated architectures. Therefore, the ceiling effect is a consequence of the evaluation protocol rather than an intrinsic property of the task itself. It follows that under random partitioning the composite scoring function (Equation (1)) is redundant, because any weighting returns YOLOv8s, and the selection framework becomes genuinely discriminative only once the leakage is removed (Section 4.5). Extending the analysis to multisite datasets is necessary to establish whether the ordering observed under D3 generalizes beyond this deployment.

This performance can be explained by three methodological factors: (i) initialization with weights pre-trained on COCO, which provided robust visual representations prior to **fine-tuning**; (ii) the homogeneity of the capture setting, achieved by using a fixed camera with a constant perspective of the same parking lot; and (iii) the augmentation of data designed to replicate environmental conditions typical of the Andean highlands, such as changes in brightness, shadows, and noise.

Notably, the **unavailable** class, although representing only 6.66% of the annotated instances, achieved the highest mAP across all architectures. Because the overall mAP corresponds to the macro-average across classes, the high performance of this minority category increases the aggregate value; therefore, the metrics disaggregated by class (Table S5) more accurately describe the performance of each category. This class comprises only 146 instances in the test set (out of 2045 in total); therefore, the lack of variation in the “mAP@0.5” across the ten independent seeds is consistent with a small and visually homogeneous sample rather than with anomalous saturation or a possible information leak.

A gap of more than ten percentage points separates the detection figures obtained on the test partition from the per-slot accuracy measured in deployment, and it requires explanation rather than being left as an apparent inconsistency. These two quantities measure different tasks in the study. The mAP evaluates the localization and classification of bounding boxes against the annotated ground truth and credits a detection whose box overlaps the reference above the IoU threshold with a correct label. Per-slot accuracy evaluates the final state assigned to a physical parking slot after the detection output has passed through the box-to-slot assignment by geometric overlap, ByteTrack identifier association, and temporal smoothing over a 25-frame window. Therefore, a frame can yield near-perfect detection metrics and still produce an incorrect slot state if a correctly detected box is assigned to an adjacent slot, an identifier is reassigned across frames, or the smoothing window retains a stale state through a transition. The converse also occurs: temporal smoothing recovers slot states from isolated detection failures that mAP penalizes.

Consequently, he size and direction of the gap are informative. Under the disjoint partition, the detector reached 0.8828 in mAP@0.5:0.95, whereas the deployed system reached 87.5% per-slot accuracy in daytime sessions, indicating that the operational bottleneck of this class of system is not located in the detector alone. The assignment and stabilization stages that convert detections into slot states carry a comparable share of the error budget, and they are the stages in which a purely detection-oriented benchmark is left unmeasured. This has a direct methodological implication for the literature: comparative studies that report only offline detection metrics—the majority of those surveyed in Section 2—characterize one component of a pipeline whose end-to-end accuracy they do not observe, and improvements in mAP cannot be assumed to transfer proportionally to the deployed system.

Table 16 places these results in the context of recent studies on parking space detection.

These results are comparable to, and even better than, those reported in recent studies on parking space detection using YOLO architectures, which reported mAP@ 0.5 ranging from 0.85 to 0.97 using datasets such as PKLot and custom university datasets [17,18,25]. The consistent convergence observed across the ten independent runs of each architecture, reflected in the narrow bands of the validation curves (Figure 4), supports the stability of the training protocol and shows no signs of overfitting within the evaluated experimental conditions.

Despite the consistency in detection metrics, the analysis of computational efficiency revealed substantial and statistically significant differences between architectures (H=47.06, p<0.001, $\varepsilon^{2}=0.96$). YOLOv8s demonstrated the highest throughput (205.49 FPS [200.93,210.05]) and the lowest p95 latency (5.24 ms [5.10,5.37]), positioning itself as the most suitable architecture for real-time vehicle monitoring applications under the defined operational constraints of ≥25 FPS and ≤40 ms. This result is consistent with recent comparisons between modern versions of YOLO and RT-DETR, where the downscaled YOLO variants typically maintain significant advantages in inference speed over larger-scale architectures [13,14].

The fact that all five architectures satisfy the nominal constraints of ≥25 FPS and ≤40 ms deserves a qualification, because it would otherwise imply that throughput beyond that threshold carries no value and that the selection should be decided on accuracy alone—under the disjoint partition, in favor of Faster R-CNN. However, the thresholds apply to the *complete* pipeline rather than to the detector in isolation, and detection is only one stage. The integrated system operates at 17 FPS, that is, a budget of approximately 58.8 ms per frame shared between detection, ByteTrack association, geometric occupancy analysis, temporal smoothing over a 25-frame window, and transmission to the web client. Within that budget YOLOv8s consumes 5.24 ms of p95 latency and leaves roughly 53.6 ms for the remaining stages; Faster R-CNN consumes 37.91 ms, which is 7.2 times more, and leaves under 21 ms—less than half. Therefore, a detector that satisfies the constraint in isolation does not guarantee that the deployed system will satisfy it, and the headroom left for the non-detection stages becomes an operative criterion. This is the sense in which the efficiency advantage of YOLOv8s remains decisive even though Faster R-CNN is nominally within limits, and it also indicates that the throughput bottleneck of this class of systems lies outside the detector.

Notably, YOLOv8s’ the higher composite performance (S=1.0000) did not stem solely from marginal differences in detection metrics but rather from its ability to combine high precision with superior computational efficiency. Faster R-CNN achieved perfect *recall* (1.0000) and, under the native per-framework thresholds, the lowest apparent *precision* among the evaluated models (0.9742±0.0228). As established in Table 10, both figures are artifacts of the evaluation configuration rather than properties of the architecture, and under harmonized thresholds, the Faster R-CNN model attains the highest precision of the five models. Although RT-DETR-L incorporates transformer-based attention mechanisms and an *end-to-end* detection design [11], it exhibits a considerably lower throughput (41.07 FPS [40.63,41.51]) than the YOLO-s variants, reducing its operational margin in scenarios with strict computational constraints. Faster R-CNN, as a two-stage detector, exhibited the highest p95 latency (37.91 ms [36.85,38.97]) and the lowest FPS (26.97[26.42,27.52]) among the evaluated models, remaining within than operational thresholds but with the smallest margin for coping with variations in than computational load. The YOLOv12s exhibited high variability in efficiency metrics (${\mathrm{SD}}_{\mathrm{FPS}}=26.85$), attributable to variable GPU load conditions during evaluation, which represents a practical limitation for production environments that require predictable latency.

The analysis by class showed consistent behavior across the architectures. The **unavailable** category achieved the best performance in all models, whereas **free** recorded the lowest values for mAP@0.5:0.95. This difference can be attributed to the visual characteristics of the classes. **Unavailable** spaces exhibit easily distinguishable patterns, such as traffic cones, marked areas, and obvious occlusions that reduce ambiguity during detection. In contrast, the *free* class is subject to greater visual variability owing to shadows cast by nearby vehicles, reflections on the pavement, and changes in daylight conditions, factors that are particularly pronounced in the high-altitude Andean environment, where solar radiation creates intense contrasts [20]. Faster R-CNN was the only architecture to achieve a mAP@0.5 of 1.0000 and a **precision** of 1.0000 in the **unavailable** class, suggesting that its two-stage detection scheme, supported by ROIAlign, is particularly effective when regions exhibit well-defined visual features. In contrast, the greater variability observed in the **precision** of the **occupied** class (0.9576±0.0342) reflects lower stability across runs, possibly related to the RPN’s sensitivity to changes in perspective and partial occlusions typical of the Andean university environment [20,21].

The perfect *recall* (1.0000±0.0000) initially obtained by Faster R-CNN across the ten independent runs stemmed from a configuration difference and did not reflect the inherent advantage of the architecture. The torchvision implementation uses a default score threshold of score_thresh = 0.05, which is considerably lower than the confidence threshold used by the Ultralytics architectures (conf = 0.25); This configuration retains virtually all proposals generated by the RPN, favoring high *Recall* at the expense of apparent *Precision*. Rather than leaving this as a caveat on the comparison, the evaluation was repeated for all five architectures with a single framework-independent evaluator at harmonized thresholds (Table 10). Under this protocol, the asymmetry disappears: Faster R-CNN attains a precision of 0.9977 [0.9955,0.9995] against 0.9959 [0.9927,0.9986] for YOLOv8s, with overlapping confidence intervals; therefore, the two architectures are statistically indistinguishable in detection quality for this task. The precision disadvantage reported in Table 9 is therefore a property of the native evaluation configuration and not of the two-stage design itself.

The practical implications of this system extend across distinct stakeholder groups. Drivers benefit directly from reduced search times because real-time occupancy information enables targeted navigation toward available slots, eliminating repeated entry and exit attempts that contribute to congestion at parking facility entrances. Facility managers can use this tool to monitor peak-demand periods, plan maintenance windows, and compute turnover rates without requiring manual surveys. University administrators can integrate occupancy data into sustainability reporting and smart campus initiatives, given that parking management has been identified as a tractable lever for reducing vehicular emissions in campus environments. Finally, at the municipal and urban planning scales, disaggregated occupancy time series from multiple facilities can support demand-responsive pricing models and inform transport infrastructure decisions [5]. However, these benefits are, contingent on successful deployment beyond the single-site controlled conditions of this study. Further discussion of the constraints on generalization is provided in the Limitations section.

The scalability of the proposed approach beyond a single university parking facility requires explicit consideration. In the current deployment, a single fixed camera covers 15 slots; urban-scale adoption would require covering hundreds to thousands of slots distributed across multiple parking lots, streets, and multi-story structures with diverse geometric configurations, camera perspectives, and occlusion patterns. Each new camera installation requires an independent polygon boundary definition, and the trained model likely requires domain adaptation through fine-tuning on locally collected images to maintain accuracy under different camera heights, lens angles, and vehicle populations. The network infrastructure for streaming multiple RTSP feeds simultaneously, together with server-side resources to run real-time inference at 17 FPS per camera, represents a non-trivial operational cost that scales linearly with the number of monitored cameras. These constraints do not preclude urban deployment, but they indicate that direct transferability of the single-site system without architectural modifications, such as edge computing offloading, federated model adaptation, or automated camera calibration pipelines, should not be assumed. Addressing these scalability dimensions is a priority for future research.

Validation of the integrated YOLOv8s + ByteTrack + FastAPI + Next.js 14 system under real-world conditions produced a nominally higher *Slot Accuracy* in the nighttime session (91.8%[89.0,94.0]) than in the daytime sessions (87.5%[85.2,89.5]). The two confidence intervals overlap; therefore, the difference is not statistically distinguishable at the α=0.05 level, and no claim of superior nighttime performance is made. One tentative explanation for the nominal gap is the greater uniformity of artificial illumination during the nighttime session compared to the dynamic shadows, reflections, and overexposed areas produced by natural sunlight during the day [20,25]. Because the nighttime result rests on a single 31-min session, this interpretation remains preliminary. Nevertheless, the error profiles differ in one respect: the intervals do support. During daytime, a lower FPR (1.6%[0.3,8.3]) and a higher FNR (13.3%[11.2,15.7]) were observed, indicating a greater tendency to fail to detect occupied parking spaces under variable lighting. Nighttime sessions recorded a higher FPR (9.3%[5.0,16.7]) and a lower FNR (7.9%[5.5,11.1]), suggesting spurious detections associated with the reflections from artificial lighting on the pavement. The FPR intervals overlap, and the corresponding difference should not be regarded as established; the FNR intervals do not, so the higher daytime false-negative rate is the one contrast supported by the data.

## 6. Limitations

Although the results support the feasibility of the proposed approach, this study has several limitations that should be considered when interpreting its results.

The main methodological limitation of the original evaluation protocol was the dataset partitioning strategy. The 70/20/10 split was performed randomly using Roboflow, without separation by capture day or session. Because the corpus was obtained using a fixed camera in a single parking lot, visually near-identical images, including scenes containing the same parked vehicles, were distributed across the training, validation, and test subsets. This limitation is no longer applicable to the present study. Section 4.4 quantifies it directly through a perceptual-hashing audit, which establishes that 50.2% of the test images under the original random split shared a 15-min acquisition window with training images, and Section 4.5 re-estimates every architecture under the fully disjoint partition D3. What remains is a narrower residual limitation: D3 rests on a single pair of camera viewpoints at one site; therefore, it bounds the magnitude of the leakage effect for this deployment without establishing how large that effect would be under other site geometries or camera configurations.

Under random partitioning it was assumed that this limitation did not affect the *relative* comparison between architectures, since all were trained and evaluated under the same protocol and were subject to the same bias. Section 4.5 shows that this assumption does not hold: the ordering obtained under D3 is essentially the inverse of the ordering obtained under R0 (Spearman ρ=−0.80), and the Pareto front expands from a single architecture to four. Therefore, both the *absolute* mAP values obtained under random partitioning and the ranking derived from them must be read as protocol-dependent quantities rather than as estimates of generalization ability under cameras, parking lots, or environmental conditions that differ from those observed during training. The validation of the integrated system under real-world operational conditions (Section *Experimental Setup*), based on direct human observation rather than a partitioned test set, provides complementary evidence that is independent of the partitioning protocol.

Another limitation of this study the origin of the dataset. The images were captured on a single university campus in Juliaca (Puno region, Peru), which is located 3824 m above sea level. Although this dataset is valuable for incorporating environmental conditions typical of a high-altitude setting, its geographical specificity may limit the direct transferability of the models to parking lots with different geometries, other types of vehicles, varying occupancy densities or contrasting weather conditions. Consequently, the external validity of the findings is more representative of similar university settings than diverse urban environments.

The comparison between Faster R-CNN (∼41 M parameters) and the YOLO-s variants (∼11 M parameters) is also not isoparametric. Therefore, the observed differences in efficiency reflect both the characteristics of each architectural paradigm and the effect of model size. The difference in decision thresholds between frameworks (score_thresh = 0.05 in torchvision versus conf = 0.25 in Ultralytics) was eliminated by re-evaluating all five architectures with a single framework-independent evaluator at harmonized thresholds (Table 10); a residual confound nevertheless remains, since the Ultralytics models were trained with the default online augmentation pipeline (mosaic, HSV jitter, random horizontal flip) whereas the torchvision Faster R-CNN was trained with horizontal flipping only, so the comparison is harmonized at evaluation time but not at training time. In addition, the composite scoring function (Equation (1)) prioritizes the inference FPS in isolation (*batch* = 1), a metric that does not correspond to the performance of the complete integrated system (17 FPS), which includes ByteTrack, geometric occupancy calculation, and WebSocket transmission. Consequently, the selection of an optimal model should consider this difference.

The integrated system was validated using only two daytime sessions and one nighttime session, which is a limited sample set for characterizing the behavior of the system under the full range of possible environmental conditions.

The evaluation also did not include adverse weather conditions, such as heavy rain, fog, or snow, which are common in real-world applications and could potentially degrade the performance of vision-based systems in practice. Similarly, the geometric method used to determine occupancy could lose accuracy in multi-level parking garages, tandem parking configurations, or situations involving significant vehicle overlap.

Finally, the computational efficiency metrics for YOLOv12s and RT-DETR-L showed variability across the runs, which can be attributed to fluctuations in GPU load during evaluation. This behavior poses a practical challenge to ensuring stable and predictable performance in production environments.

Ano-ther limitation inherent to the system’s design is its dependence on a fixed camera with a constant viewpoint and predetermined field of view. In this configuration, parking slot polygon boundaries are manually defined once for a specific camera position and must be reconfigured if the camera is repositioned, replaced, or if the zoom or tilt settings change. This is a structural constraint that differentiates camera-based monitoring from per-slot sensor approaches, which are invariant to camera placement. Additionally, because the model was trained on images from a single camera angle, it encoded perspective-specific visual features; deployment under substantially different camera heights, angles, or focal lengths would likely require retraining on data collected using the new configuration.

The comparative evaluation includes five specific architectures (YOLOv8s, YOLOv11s, YOLOv12s, RT-DETR-L, Faster R-CNN), but does not cover several architectures that have been applied to parking and vehicle-detection scenarios in the period since the study was designed—particularly YOLOv9, which introduced Programmable Gradient Information and Generalized Efficient Layer Aggregation Networks, and YOLOv10, which eliminates non-maximum suppression through one-to-one head matching to reduce post-processing latency [7]. YOLO-NAS, which is based on a neural architecture search for speed-accuracy optimization, was also not included. The selection of the evaluated architectures reflects the practical constraints of running ten independent training runs per model, not a judgment on the merits of the excluded architectures. Future studies applying the same homogeneous protocol to these models would provide a more complete survey of the current detection landscape for parking monitoring applications.

The integrated system was validated using only two daytime sessions and one nighttime session, each lasting 31 min, for a total of 93 min of operation. This duration covers only a narrow window of potential conditions: transitional lighting at dawn and dusk, adverse weather (rain, fog, snow), atypical occupancy patterns during campus events, and seasonal variations in ambient light and temperature. Therefore, the validation results should be interpreted as indicative of system performance under stable, clear-sky conditions characteristic of the Andean dry season at the study site, and not as a robust characterization of performance across the full operational envelope. Furthermore, the ground truth was recorded at one-minute intervals, a resolution that cannot capture occupancy changes occurring within a minute, such as vehicles parking or leaving between the recording intervals.

The analysis does not include a systematic characterization of failure modes or edge cases beyond the aggregate FPR and FNR metrics. The inspection of false-negative (115 missed occupied slots in the daytime sessions) and false-positive cases (9 spurious detections in the nighttime session) indicated that errors were not uniformly distributed across conditions. Missed detections were disproportionately associated with partial occlusions by pedestrians, irregular shadows from overhead structures, and transitional occupancy states occurring between consecutive ground-truth recording intervals. Nighttime false positives were related to specular reflections from infrared illumination on metallic vehicle surfaces. Rigorous failure-mode analysis requiring frame-level annotation of error cases was outside the scope of this study but is important for operational deployment, where a concentration of errors in specific environmental conditions could undermine user trust and reduce the practical utility of the real-time occupancy display.

Finally, the scope of the reported performance was limited to daytime and nighttime clear-sky conditions at a single site, observed from a fixed-camera viewpoint. The cross-illumination experiment indicated that performance transfers across the day/night transition at this site (0.9913 to 0.9880 in mAP@0.5:0.95), but no claim was made regarding adaptability to environmental variability in general, and none of the sessions included adverse weather conditions. References to variable lighting conditions in this study should be understood as referring to daytime/nighttime contrast under clear skies.

Finally, the ranking reversal reported in Section 4.5 was established using a single dataset and pair of camera viewpoints. Whether two-stage detectors systematically generalize better across viewpoints than single-stage detectors or whether this reflects a particularity of this deployment, cannot be settled with the present evidence and would require replication across sites and camera configurations.

## 7. Future Work

Based on the limitations identified, several lines of research are proposed for future studies.

One approach is to expand the dataset by incorporating multiple geographic locations, different types of cameras, and adverse environmental conditions such as heavy rain, dense fog, snow, or direct glare. This would allow us to evaluate the generalization ability of the models and increase their robustness in scenarios that are not represented in the current dataset.

Lightweight optimization techniques, such as quantization and model pruning, should also be explored to facilitate the deployment of low-power edge devices. This strategy helps mitigate the computational variability observed in large-scale architectures and promotes system scalability in resource-constrained environments [26,27].

Another line of research involves incorporating richer semantic information or instance segmentation methods to address more complex parking scenarios, such as overlapping vehicles or multi-level parking garages, thereby overcoming the limitations of an approach based solely on bounding boxes.

Validation on external benchmarks, such as PKLot or CNRPark-EXT, would establish whether the methodology can be transferred across sites. Because the present annotations describe parking slots rather than vehicles, such an evaluation requires re-annotation rather than direct transfer of the trained weights, and is therefore left for future work.

Finally, the proposed system was expanded with predictive capabilities by integrating historical occupancy data and real-time traffic flow data. This would make it possible to anticipate parking availability and broaden the system’s utility for urban mobility planning and the development of smart-city initiatives.

## 8. Conclusions

This study compared five object detection architectures, YOLOv8s, YOLOv11s, YOLOv12s, RT-DETR-L, and Faster R-CNN, for intelligent vehicle occupancy monitoring on a university campus in Juliaca (Puno region), Peru, located at 3824 m a.s.l. Based on the obtained results, the following conclusions can be highlighted.

The generalization performance of the evaluated architectures was characterized by the fully disjoint partition D3, under which mAP@0.5:0.95 ranged from 0.9325 for Faster R-CNN to 0.8763 for YOLOv11s. These estimates should inform any deployment decisions. The values obtained under random partitioning—all above 0.985 and within 0.0055 of one another—reflect a protocol in which visually near-identical scenes appear in both the training and test subsets. They are reported here to quantify this effect, not as performance claims.

The difference between the two protocols is not merely a matter of magnitude. The ranking of the five architectures under random partitioning was essentially the inverse of the ranking under disjoint partitioning (ρ=−0.80), and the Pareto front expanded from a single architecture to four. Therefore, a leakage-affected protocol does more than inflate the reported figures; it removes the information that would allow architectures to be distinguished and can reverse the resulting recommendation.

Among the analyzed architectures, YOLOv8s offers the most favorable balance between accuracy and computational efficiency for real-time deployment, reaching 205.49 FPS with a p95 latency of 5.24 ms and comfortably exceeding the operational requirements established for this application (≥25 FPS and ≤40 ms). Under the disjoint partition, it is the fastest of the four non-dominated architectures and is selected in 73.3% of the weightings examined, although it ranks fourth in accuracy: Faster R-CNN attains 0.9325 against 0.8828, at between one-fifth and one-eighth of the throughput. Therefore, the recommendation of YOLOv8s rests on computational efficiency rather than detection accuracy, a distinction that only becomes visible once the partitioning protocol removes leakage. The efficiency remains decisive even though every architecture satisfies the nominal constraints of ≥25 FPS and ≤40 ms, because those constraints apply to the complete pipeline: at the 17 FPS operating point of the integrated system, YOLOv8s leaves approximately 53.6 ms of the per-frame budget for tracking, occupancy geometry, temporal smoothing and transmission, whereas Faster R-CNN leaves under 21 ms. Where accuracy is the priority and the deployment tolerates a lower refresh rate, Faster R-CNN is the better choice on the evidence reported here. The Kruskal–Wallis analysis (H=47.06, p<0.001) confirmed significant differences in computational efficiency among architectures; however, YOLOv8s and YOLOv11s showed no statistically significant difference in FPS between themselves; both achieved significantly higher FPS than RT-DETR-L and Faster R-CNN, whereas YOLOv11s did not differ significantly from YOLOv12s in the Dunn–Bonferroni pairwise comparison.

The per-class analysis revealed consistent results. The **unavailable** category was the easiest to detect for all architectures, while **free** recorded the lowest mAP@0.5:0.95 values, mainly because of the visual variability associated with shadows, reflections and lighting changes characteristic of the high-altitude Andean environment. The precision disadvantage initially attributed to Faster R-CNN proved to be an artifact of the evaluation protocol rather than a property of the architecture. Under a single framework-independent evaluator with harmonized decision thresholds, Faster R-CNN attained a precision of 0.9977 [0.9955,0.9995] against 0.9959 [0.9927,0.9986] for YOLOv8s, with overlapping confidence intervals. The two architectures are statistically indistinguishable in detection quality for this task; the selection of YOLOv8s rests entirely on computational efficiency, where it is 7.6 times faster.

Validation of the integrated system YOLOv8s + ByteTrack + FastAPI + Next.js 14 under real operating conditions achieved a *Slot Accuracy* of 87.5% [85.2,89.5] during daytime sessions and 91.8% [89.0,94.0] in the nighttime session. The confidence intervals for the two conditions overlap, so the difference is not statistically distinguishable. Combined with the fact that the nighttime figure rests on a single 31-min session, no claim of superior nighttime performance is made.

By reducing the time spent searching for available parking spaces, the proposed system contributes to mitigating the **cruising** phenomenon that motivated this study. This study also provides a systematic comparison of single-stage CNN detectors, **transformer**-based models, and two-stage detectors under real conditions in an Andean university environment. In future work, it is pertinent to extend the evaluation to multiple geographic locations, incorporate adverse weather scenarios such as rain and fog, and explore low-power **edge** architectures [26,27] that favor system scalability in university environments with limited computational resources.

## Figures and Tables

**Table 1:** Comparison of indexed related studies on intelligent parking and vehicle detection.

Author/Year	Objective	Model Used	Main Results	Limitations	Relationship to This Study
Rafique et al. (2023) [6]	Develop an optimized intelligent parking management system using deep learning.	YOLOv5s	Implemented vehicle detection on the PKLot dataset and real operational scenarios, obtaining suitable results for real-time occupancy monitoring.	Sensitivity to lighting variations, camera position, and image quality. Single-paradigm study (one-stage CNN only); no cross-paradigm comparison with transformer-based or two-stage detectors.	Supports the use of YOLO detectors for scalable intelligent parking systems.
Saputra et al. (2024) [15]	Detect vehicles and parking space availability in intelligent parking environments.	YOLOv5	Validated the application of YOLOv5 for vehicle detection and automatic occupancy estimation.	Performance decreases under occlusions and environmental variability. Single-paradigm study; no cross-paradigm comparison or computational efficiency metrics reported.	Provides experimental evidence supporting the use of YOLO for parking detection.
da Luz et al. (2026) [7]	Evaluate recent YOLO architectures using region-of-interest selection and edge/cloud processing.	YOLOv8, YOLOv9, YOLOv10, YOLOv11	Achieved high balanced accuracy levels in real intelligent parking scenarios.	Performance depends significantly on the capabilities of the hardware used. Restricted to the YOLO family; excludes transformer-based (RT-DETR) and two-stage (Faster R-CNN) paradigms from comparison.	Serves as a direct reference for comparisons among recent YOLO versions.
Ren et al. (2017) [12]	Propose an object detection architecture based on integrated region proposals.	Faster R-CNN (ResNet-50 FPN)	Achieved high detection accuracy through region refinement and pyramid feature extraction.	Presents higher computational costs and lower inference speed than one-stage detectors. Architecture proposed as a standalone contribution; not benchmarked against one-stage CNN or transformer-based detectors in parking-specific scenarios.	Serves as a high-accuracy reference for comparing the robustness and accuracy of vehicle detection.
Zhao et al. (2024) [11]	Introduce a real-time transformer-based object detector.	RT-DETR	Achieved competitive accuracy and real-time performance on COCO using end-to-end detection.	Requires greater computational resources than lightweight CNN-based detectors. Evaluated on COCO; not applied to parking detection; no direct comparison with one-stage CNN detectors in domain-specific conditions.	Provides a modern transformer-based approach for experimental comparison.
Zunair et al. (2024) [16]	Introduce a geographically diverse road-scene dataset and benchmark object detectors on it.	YOLO variants, DETR family	Showed that detectors trained on geographically homogeneous data generalize poorly to different locations.	Driving-perspective road scenes, not fixed-camera parking monitoring; generalization addressed through dataset diversity rather than partitioning protocol.	Independently documents the generalization gap that this study quantifies through leakage-controlled partitioning.

**Table 2:** Comparative configuration of the evaluated architectures.

Model	Type	Parameters	Framework	Group
YOLOv8s	One-stage CNN	∼11 M	Ultralytics 8.4	Primary
YOLOv11s	One-stage CNN	∼10 M	Ultralytics 8.4	Primary
YOLOv12s	CNN + Attention	∼11 M	Ultralytics 8.4	Primary
RT-DETR-L	One-stage Transformer	∼32 M	Ultralytics 8.4	Reference
Faster R-CNN ResNet50-FPN	Two-stage CNN	∼41 M	torchvision 0.21	Reference

**Table 3:** Criteria used for optimal model selection.

Metric	Objective	Weight	Constraint
mAP@0.5:0.95	Maximize	0.60	–
FPS	Maximize	0.25	≥25 FPS
p95 Latency	Minimize	0.15	≤40 ms

**Table 4:** Dataset split and augmentation.

Subset	Raw Images	After Augmentation	Proportion (%)
Training	1024	3072	70
Validation	293	293	20
Test	146	146	10
**Total **	**1463**	**3511**	**100**

Bold indicates total values.

**Table 5:** Leakage indicators under the original and the revised partitions.

Indicator	Original (Random)	T2	T1
Acquisition days shared between training and test	20	0	0
Minimum test-to-training separation	0 s	6728 s	352,681 s
Median test-to-training separation	899 s	54,918 s	580,383 s
Test images within 15 min of a training image	50.2%	0%	0%

**Table 6:** Instance counts by class and subset, at file level and at source level.

Level	Subset	Images	Free	Unavailable	Occupied	Total
File	Training	3072	26,958	3072	16,050	46,080
File	Validation	293	2354	292	1748	4394
File	Test	146	1208	146	836	2190
File	**Total **	**3511**	**30,520**	**3510**	**18,634**	**52,664**
Source	Training	1024	8986	1024	5350	15,360
Source	Validation	293	2354	292	1748	4394
Source	Test	146	1208	146	836	2190
Source	**Total**	**1463**	**12,548**	**1462**	**7934**	**21,944**

File-level counts include the three augmented copies generated for each training image, and the source-level counts refer to distinct captured scenes. The test subset contained 2190 instances, of which 2044 belonged to the *free* and *occupied* classes, respectively. Bold indicates total values.

**Table 7:** Overall detection performance for the five architectures (mean ± SD and 95% CI over 10 independent runs).

Model	P [95% CI]	R [95% CI]	F1 [95% CI]	mAP@0.5 [95% CI]	mAP@0.5:0.95 [95% CI]
YOLOv8s	0.9964±0.0015 [0.9953,0.9974]	0.9971±0.0009 [0.9965,0.9978]	0.9967±0.0009 [0.9961,0.9974]	0.9948±0.0002 [0.9946,0.9949]	0.9916±0.0003 [0.9913,0.9918]
YOLOv11s	0.9964±0.0009 [0.9958,0.9970]	0.9975±0.0007 [0.9971,0.9980]	0.9970±0.0005 [0.9966,0.9973]	0.9947±0.0001 [0.9946,0.9947]	0.9910±0.0004 [0.9907,0.9913]
YOLOv12s	0.9963±0.0008 [0.9958,0.9969]	0.9975±0.0006 [0.9971,0.9979]	0.9969±0.0004 [0.9967,0.9972]	0.9946±0.0001 [0.9945,0.9946]	0.9906±0.0003 [0.9903,0.9908]
RT-DETR-L	0.9962±0.0008 [0.9956,0.9967]	0.9972±0.0007 [0.9967,0.9977]	0.9967±0.0004 [0.9964,0.9970]	0.9946±0.0002 [0.9945,0.9947]	0.9907±0.0006 [0.9903,0.9912]
Faster R-CNN	0.9742±0.0228 [0.9579,0.9905]	1.0000±0.0000 [1.0000,1.0000]	0.9868±0.0118 [0.9783,0.9953]	0.9935±0.0010 [0.9928,0.9942]	0.9858±0.0042 [0.9828,0.9888]

All metrics are reported as mean ± SD [95% CI] over 10 independent runs. with seeds {42,123,456,789,1000,1234,2024,2025,3141,9999}. The 95% CI for detection metrics was estimated via *bootstrap resampling* (B=1000) on the test set. The mean ± SD was computed over 10 independent runs to report inter-run variability.

**Table 8:** Computational efficiency metrics of the five architectures (mean ± SD [95% CI]).

Model	FPS [95% CI]	Lat p50 (ms) [95% CI]	Lat p95 (ms) [95% CI]	GPU MB [95% CI]
YOLOv8s (n=10)	205.49±6.38 [200.93,210.05]	4.77±0.14 [4.66,4.87]	5.24±0.19 [5.10,5.37]	4093±333 [3855,4331]
YOLOv11s (n=10)	161.20±5.74 [157.09,165.31]	6.12±0.20 [5.98,6.26]	6.83±0.85 [6.23,7.44]	4307±3 [4305,4309]
YOLOv12s (n=10)	94.24±26.85 [75.03,113.45]	11.85±4.68 [8.50,15.20]	12.84±5.61 [8.83,16.85]	4675±1609 [3524,5825]
RT-DETR-L (n=10)	41.07±0.62 [40.63,41.51]	24.19±0.40 [23.91,24.48]	25.53±1.05 [24.78,26.28]	6216±1259 [5316,7117]
Faster R-CNN (n=10)	26.97±0.76 [26.42,27.52]	37.09±1.00 [36.38,37.81]	37.91±1.48 [36.85,38.97]	2968±4 [2965,2970]

YOLOv12s and RT-DETR-L exhibited high inter-run variability in the GPU MB, which was attributable to the variable GPU load conditions during evaluation. The Faster R-CNN GPU MB with minimal variability (±4 MB) indicated static allocation during inference.

**Table 9:** Results of the composite scoring function for the evaluated architectures.

Model	mAP@0.5:0.95	FPS	Lat p95 (ms)	$\tilde{\mathrm{mAP}}$	$\tilde{\mathrm{FPS}}$	$1-\tilde{\mathrm{Lat}}$	Score *S*	Rank
YOLOv8s (n=10)	0.9916	205.49	5.24	1.0000	1.0000	1.0000	1.0000	#1
YOLOv11s (n=10)	0.9910	161.20	6.83	0.9048	0.7519	0.9512	0.8736	#2
YOLOv12s (n=10)	0.9906	94.24	12.84	0.8235	0.3768	0.7674	0.7034	#3
RT-DETR-L (n=10)	0.9907	41.07	25.53	0.8547	0.0790	0.3790	0.5894	#4
Faster R-CNN (n=10)	0.9858	26.97	37.91	0.0000	0.0000	0.0000	0.0000	#5

Weighting: mAP@0.5:0.95 =0.6; FPS =0.25; $(1-\tilde{{\mathrm{Lat}}_{p95}})=0.15$. Min–Max normalization was applied to the five evaluated models. Operational thresholds: FPS ≥25 and p95 latency ≤40 ms; all models satisfied them. Faster R-CNN obtained a score =0.0000 because it had the lowest normalized mAP and FPS scores and the highest p95 latency, resulting in a normalized inverse-latency score of 0.0000. This reflects the lowest position in normalized space, not a null performance.

**Table 10:** Unified evaluation with harmonized decision thresholds (146 test images, 2190 instances; bootstrap 95% CI, B=1000).

Model	mAP@0.5:0.95	mAP@0.5	Precision	Recall	F_1_
YOLOv8s	0.9896	0.9931	0.9959 [0.9927,0.9986]	1.0000	0.9979
Faster R-CNN	0.9886	0.9958	0.9977 [0.9955,0.9995]	1.0000	0.9989

**Table 11:** Detection performance under six partitioning protocols (mean ± SD across independent seeds).

Partition	Controls for	Seeds	YOLOv8s	YOLOv11s
R0 (control)	—	3	0.9913±0.0004	0.9910±0.0003
T2	Temporal leakage	3	0.9889±0.0003	0.9875±0.0003
D1	Illumination	3	0.9880±0.0014	0.9879±0.0005
T1	Temporal leakage and drift	3	0.9828±0.0041	0.9793±0.0020
D2	Viewpoint geometry	10	0.9737±0.0073	0.9573±0.0086
D3	Geometry and time	10	0.8828±0.0202	0.8763±0.0247

Values are mAP@0.5:0.95 on the test subset of each partition.

**Table 12:** Decomposition of the degradation into its components (YOLOv8s).

Component	Effect on mAP@0.5:0.95
Temporal separation in isolation (R0 → T1)	0.0085
Viewpoint separation in isolation (R0 → D2)	0.0176
Sum of the individual effects	0.0261
Both applied jointly (R0 → D3)	0.1085
Interaction term, *I* (joint − sum)	+0.0824
Superadditivity factor, *F* (joint/sum)	4.16

*I* and *F* are two distinct summaries of the same interaction: *I* is expressed in mAP units and *F* is expressed as a dimensionless ratio. Both are derived from Equations (5)–(8).

**Table 13:** Validation metrics of the integrated system under daytime and nighttime lighting conditions.

Metric	Daytime [95% CI]	Nighttime [95% CI]
Total observations	930	465
Slot Accuracy (%)	87.5 [85.2,89.5]	91.8 [89.0,94.0]
False Positives (count)	1	9
False Negatives (count)	115	29
False Positive Rate (%)	1.6 [0.3,8.3]	9.3 [5.0,16.7]
False Negative Rate (%)	13.3 [11.2,15.7]	7.9 [5.5,11.1]

The 95% CI was calculated using the Wilson method for proportions. Daytime: Two sessions, 930 observations. Nighttime: 1 session and 465 observations. FPR is calculated over free slots; FNR over observed occupied slots.

**Table 14:** Architecture ranking under random and disjoint partitioning (mean ± SD over ten seeds).

Model	mAP@0.5:0.95 (R0)	Rank	mAP@0.5:0.95 (D3)	Rank
Faster R-CNN	0.9858	5	0.9325±0.0105	**1**
RT-DETR-L	0.9907	3	0.9072±0.0267	2
YOLOv12s	0.9906	4	0.9050±0.0183	3
YOLOv8s	0.9913	1	0.8828±0.0202	4
YOLOv11s	0.9910	2	0.8763±0.0247	5

Spearman’s rank correlation between both protocols is ρ=−0.80.

**Table 15:** Selection frequency over 10,000 weight vectors sampled uniformly from the simplex.

Model	Pareto (R0)	Pareto (D3)	Selected (R0)	Selected (D3)
Faster R-CNN	dominated	non-dominated	0.0%	20.4%
RT-DETR-L	dominated	non-dominated	0.0%	0.0%
YOLOv12s	dominated	non-dominated	0.0%	6.3%
YOLOv8s	non-dominated	non-dominated	100.0%	73.3%
YOLOv11s	dominated	dominated	0.0%	0.0%

Min–max normalization. Repeating the study under ratio and *z*-score normalizations yielded 99.5% and 71.2% for YOLOv8s, respectively; thus, the outcome was not an artifact of the normalization scheme.

**Table 16:** Comparison of results with related studies on vehicle occupancy detection.

Study	Model	Dataset	mAP@0.5	FPS
Rafique et al. (2023) [6]	YOLOv5s	PKLot	0.91	–
Fathurrahman et al. (2025) [17]	YOLOv8	Custom	0.94	–
Gallego et al. (2025) [25]	YOLO	University	0.97	–
da Luz et al. (2026) [7]	YOLOv11	PKLot	0.95	–
This study—YOLOv8s	YOLOv8s	Juliaca, 3824 m above sea level	0.9948	205.49
This study—YOLOv11s	YOLOv11s	Juliaca, 3824 m above sea level	0.9947	161.20

(–) FPS was not reported in the original study. Values from previous studies were obtained from the original reports of each study.

## Data Availability

The source code for model training, the detection and tracking pipeline, and the web monitoring system are publicly available at https://github.com/GaryFernandoYM/smart-parking-detection (accessed on 18 August 2026). Pre-trained model weights for all five evaluated architectures are available at https://huggingface.co/GaryFer/smart-parking-weights (accessed on 18 August 2026). The annotated dataset is publicly accessible at Roboflow Universe: https://universe.roboflow.com/fernando-qzldu/smart-parking-upeu/4 (accessed on 18 August 2026).

## Supplementary Materials

The following supporting information can be downloaded at: https://www.mdpi.com/article/10.3390/s26175329/s1. Table S1: Hardware and software environment used in the experiments; Table S2: Metrics used during the experimental validation; Table S3: Class distribution in the final dataset; Table S4: Average epoch of *early stopping* activation (mean ± SD; 10 runs per model except RT-DETR-L stopping epoch, averaged over 9 non-degenerate runs) and final validation mAP@0.5 (mean ± SD over all 10 runs); Table S5: Per-class detection performance for the five architectures (mean ± SD [95% CI] over 10 independent runs); Table S6: Pairwise FPS comparisons—Dunn–Bonferroni *post hoc* test (H=47.06, p<0.001); Table S7: Sensitivity analysis: rank 1 model under weight perturbations.

## Abbreviations

The following abbreviations are used in this manuscript:

CNN	Convolutional Neural Network
YOLO	You Only Look Once
FPS	Frames Per Second
mAP	Mean Average Precision
NMS	Non-Maximum Suppression
RPN	Region Proposal Network
FPN	Feature Pyramid Network
IoU	Intersection over Union
RTSP	Real Time Streaming Protocol
GPU	Graphics Processing Unit
SD	Standard Deviation
CI	Confidence Interval
FPR	False Positive Rate
FNR	False Negative Rate
