# Supplementary material for: Comparative Evaluation of Deep Learning Object Detectors for Real-Time Parking Occupancy Detection Under Variable Lighting Conditions
Source: Sensors (Basel). 2026 Aug 22;26(17):5329. doi: 10.3390/s26175329 (PMC13568434; doi:10.3390/s26175329)
Supplement: Supplementary file 1 [file sensors-26-05329-s001.zip › sensors-4459598-supplementary.pdf]

Supplementary Materials

Comparative Evaluation of Deep Learning Object Detectors for Real-Time Parking Occupancy Detection under Variable Lighting Conditions

This document contains supporting tables referenced in the main manuscript. Table S1 describes the computational environment; Table S2 defines the metrics used in the field validation; Table S3 reports the class distribution of the dataset; Table S4 reports early-stopping behaviour across runs; Table S5 disaggregates detection performance by class; Table S6 reports the pairwise post hoc comparisons of throughput; and Table S7 reports the sensitivity of the composite score to weight perturbations.

Table S1: Hardware and software environment used in the experiments.

**Component**	**Specification / Version**
Operating system	Ubuntu 22.04.5 LTS
CPU	AMD Ryzen Threadripper PRO 5975WX (32 cores, 64 threads)
GPU	NVIDIA GeForce RTX 3080 (10 GB VRAM; 1 of 2 available GPUs used)
CUDA	12.4
Python	3.10.12
PyTorch	2.6.0+cu124
torchvision	0.21.0+cu124
Ultralytics	8.4.51
NumPy	1.26.4
Shapely	2.1.2
ByteTrack	Integrated implementation in Ultralytics (lap 0.5.13)

Table S2: Metrics used during the experimental validation

**Category**	**Metric**	**Description**
Detection	Precision	Proportion of correct positive detections
	Recall	Ability to detect vehicles present
	F1-score	Balance between precision and recall
	mAP@0.5	Mean Average Precision at IoU = 0.5
	mAP@0.5:0.95	Mean Average Precision across IoU thresholds [0.5, 0.95]
Efficiency	FPS	Frames per second during standalone inference (*batch*=1)
	p50/p95 Latency	Median and 95th percentile inference latency
	GPU Memory (MB)	GPU memory consumption during inference
Integrated system	Per-slot accuracy	Accuracy of free/occupied classification
	FP Rate (%)	Free parking slots incorrectly classified as occupied
	FN Rate (%)	Occupied parking slots incorrectly classified as free

Table S3: Class distribution in the final dataset.

**Class**	**Instances**	**Proportion (%)**
free	30,520	57.95
occupied	18,634	35.38
unavailable	3,510	6.66
**Total**	**52,664**	**100.00**

Table S4: Average epoch of *early stopping* activation (mean ± SD; 10 runs per model except RT-DETR-L stopping epoch, averaged over 9 non-degenerate runs) and final validation mAP@0.5 (mean ± SD over all 10 runs).

**Model**	**Stopping epoch (mean ± SD)**	**Early stop**	**Final mAP@0.5**
YOLOv8s	80.0 ± 12.3	10/10	0.9948
YOLOv11s	82.8 ± 15.0	9/10	0.9947
YOLOv12s	81.0 ± 11.7	10/10	0.9946
RT-DETR-L	77.0 ± 14.1 *	10/10	0.9946
Faster R-CNN	30.1 ± 8.1	10/10	0.9935

Early stopping was triggered in 49/50 runs (98%). The final mAP@0.5 corresponds to the mean reported in the overall detection performance table of the main manuscript.
* RT-DETR-L: average computed over 9 runs; seed 42 (stopped at epoch 1) was excluded.

Table S5: Per-class detection performance for the five architectures (mean ± SD [95% CI] over 10 independent runs).

**Model**	**Class**	**mAP@0.5**	**mAP@0.5:0.95**	**P**	**R**
YOLOv8s	free	0.9945 ± 0.0005 [0.9941, 0.9948]	0.9877 ± 0.0013 [0.9868, 0.9886]	0.9968 ± 0.0027 [0.9948, 0.9987]	0.9947 ± 0.0016 [0.9935, 0.9958]
	occupied	0.9949 ± 0.0001 [0.9948, 0.9950]	0.9921 ± 0.0005 [0.9918, 0.9924]	0.9934 ± 0.0022 [0.9918, 0.9949]	0.9968 ± 0.0020 [0.9953, 0.9982]
	unavailable	0.9950 ± 0.0000 [0.9950, 0.9950]	0.9949 ± 0.0002 [0.9947, 0.9950]	0.9989 ± 0.0010 [0.9981, 0.9996]	1.0000 ± 0.0000 [1.0000, 1.0000]
YOLOv11s	free	0.9943 ± 0.0002 [0.9941, 0.9944]	0.9868 ± 0.0006 [0.9863, 0.9872]	0.9971 ± 0.0023 [0.9955, 0.9987]	0.9954 ± 0.0011 [0.9946, 0.9962]
	occupied	0.9948 ± 0.0001 [0.9947, 0.9949]	0.9915 ± 0.0006 [0.9911, 0.9919]	0.9927 ± 0.0015 [0.9916, 0.9937]	0.9972 ± 0.0015 [0.9962, 0.9983]
	unavailable	0.9950 ± 0.0000 [0.9950, 0.9950]	0.9948 ± 0.0005 [0.9944, 0.9951]	0.9995 ± 0.0009 [0.9988, 1.0001]	1.0000 ± 0.0000 [1.0000, 1.0000]
YOLOv12s	free	0.9942 ± 0.0002 [0.9940, 0.9943]	0.9856 ± 0.0009 [0.9850, 0.9862]	0.9972 ± 0.0013 [0.9963, 0.9981]	0.9951 ± 0.0010 [0.9944, 0.9957]
	occupied	0.9945 ± 0.0003 [0.9942, 0.9947]	0.9911 ± 0.0009 [0.9905, 0.9918]	0.9924 ± 0.0016 [0.9912, 0.9935]	0.9974 ± 0.0010 [0.9967, 0.9980]
	unavailable	0.9950 ± 0.0000 [0.9950, 0.9950]	0.9949 ± 0.0002 [0.9948, 0.9951]	0.9994 ± 0.0007 [0.9989, 0.9999]	1.0000 ± 0.0000 [1.0000, 1.0000]
RT-DETR-L	free	0.9940 ± 0.0005 [0.9937, 0.9943]	0.9862 ± 0.0012 [0.9854, 0.9871]	0.9964 ± 0.0015 [0.9953, 0.9975]	0.9955 ± 0.0007 [0.9950, 0.9960]
	occupied	0.9947 ± 0.0002 [0.9946, 0.9949]	0.9910 ± 0.0011 [0.9902, 0.9917]	0.9935 ± 0.0013 [0.9926, 0.9945]	0.9963 ± 0.0021 [0.9947, 0.9978]
	unavailable	0.9950 ± 0.0000 [0.9950, 0.9950]	0.9950 ± 0.0000 [0.9950, 0.9950]	0.9986 ± 0.0007 [0.9981, 0.9991]	1.0000 ± 0.0000 [1.0000, 1.0000]
Faster R-CNN	free	0.9899 ± 0.0002 [0.9898, 0.9901]	0.9802 ± 0.0051 [0.9765, 0.9838]	0.9801 ± 0.0213 [0.9649, 0.9954]	0.9977 ± 0.0015 [0.9966, 0.9987]
	occupied	0.9905 ± 0.0030 [0.9884, 0.9927]	0.9784 ± 0.0094 [0.9717, 0.9851]	0.9576 ± 0.0342 [0.9332, 0.9821]	0.9988 ± 0.0006 [0.9984, 0.9992]
	unavailable	1.0000 ± 0.0000 [1.0000, 1.0000]	0.9988 ± 0.0004 [0.9985, 0.9991]	1.0000 ± 0.0000 [1.0000, 1.0000]	1.0000 ± 0.0000 [1.0000, 1.0000]

95% CI estimated via *bootstrap resampling* ($B = 1{,}000$) on the test set.

Table S6: Pairwise FPS comparisons—Dunn–Bonferroni *post hoc* test ($H = 47.06$, $p < 0.001$).

	**YOLOv8s**	**YOLOv11s**	**YOLOv12s**	**RT-DETR-L**	**Faster R-CNN**
YOLOv8s	1.0000	1.0000	**0.0215**	**0.0000**	**0.0000**
YOLOv11s	1.0000	1.0000	1.0000	**0.0215**	**0.0000**
YOLOv12s	**0.0215**	1.0000	1.0000	1.0000	**0.0215**
RT-DETR-L	**0.0000**	**0.0215**	1.0000	1.0000	1.0000
Faster R-CNN	**0.0000**	**0.0000**	**0.0215**	1.0000	1.0000

Bold = $p < 0.05$ after Bonferroni correction.
$p = 0.0000$ values indicate $p < 0.0001$.

Table S7: Sensitivity analysis: rank 1 model under weight perturbations.

w_{mAP}	w_{FPS}	w_{lat}	**Rank 1**
0.5	0.1	0.4	YOLOv8s
0.5	0.2	0.3	YOLOv8s
0.5	0.3	0.2	YOLOv8s
0.6	0.1	0.3	YOLOv8s
0.6	0.2	0.2	YOLOv8s
0.6	0.3	0.1	YOLOv8s
0.7	0.1	0.2	YOLOv8s
0.7	0.2	0.1	YOLOv8s

YOLOv8s obtained rank 1 in all 8 evaluated configurations (100%).
